# Supplementary material for: The unequal impact of the coronavirus pandemic: Evidence from seventeen developing countries
Source: PLoS One. 2020 Oct 7;15(10):e0239797. doi: 10.1371/journal.pone.0239797 (PMC7540865; doi:10.1371/journal.pone.0239797)
Supplement: S1 File — (PDF) [file pone.0239797.s001.pdf]

Supplementary Information

Data Collection and Construction

Questionnaire

The questionnaire collects data on monthly total household income in January 2020 and expected monthly total household income in April 2020 in ranges constructed as multiples of the minimum wage in that country. The questionnaire asked if total household income was reduced during the past week. The questionnaire asked whether a household member lost their job or closed their business. The recall period for these questions was randomized between 1 week, 2 weeks, and 1 month. For respondents that remain in the labor market, the questionnaire asks whether the respondent worked outside the home or from home during the past week and we code a variable to represent teleworking if the respondent reported working from home. Using the past week as the recall period, the questionnaire asks if any member of the household went hungry due to lack of food and asks respondents to report the strength of their agreement with a statement "I am eating less healthy than normal" on a scale from 1 (complete disagreement) to 5 (complete agreement). The questionnaire asks if any member of the household received (gave) a transfer or a loan from (to) another household during the past week. The questionnaire also asks respondents to report the strength of their agreement with a statement that COVID-19 should be the top priority of the national government on a scale from 1 (complete disagreement) to 5 (complete agreement) and asked whether respondents think that non-essential businesses should close.

Sample questionnaires in English, Spanish and Dutch can be downloaded here: `https://www.dropbox.com/sh/uuv17cfaz94kw4h/AAAWvgJUNYEBHhNxZPcQVv3Za?dl=0`.

Data Construction

The questionnaire was implemented online using Qualtrics. Column (1) of Table S1 displays the launch date of the survey in each country. We construct the data set in several steps and the resulting number of observations that comprise the sample for each country is shown in column (2) of Table S1. First, we restrict the sample to completed surveys. Overall, approximately 59% of surveys that are started are completed. Second, we restrict the sample to surveys associated with IP addresses within the borders of the country for which the respondent is completing the survey. Across all countries in the sample, over 99% of completed surveys comply with this criteria. Third, Qualtrics flags surveys that are completed on the same device and likely to be repeat surveys completed by the same individual or household based on cookies. This is an imperfect filter. For example, it will not recognize repeated surveys by the same individual or household that are completed on different devices. Surveys flagged as repeats comprise less than 2.3% of completed surveys and we drop these surveys from the sample.

Recruitment Materials

Recruitment through Social Media Posts

We recruited participants for the survey using paid advertisement posts on Facebook and Instagram. To be eligible to participate in the survey, participants had to be at least 18 years old. In each country, we targeted the social media campaign to people ages 19 and up. We utilized keywords that have broad appeal in the country, such as futbol (soccer) and the names of futbol stars or local celebrities, and are unrelated to COVID-19 in order to avoid selecting respondents based on their knowledge or experience regarding the current pandemic. In countries in which the average age of our sample was high or expected to be high, we utilized a second, simultaneous social media campaign with the same images and keywords, but specifically targeted to millennials aged 24-35.

In each country, we used three posts, an image of a male wearing a face mask, an image of a female wearing a face mask, and an image of an iconic landmark in that country. The posts were adapted to each country by changing the image of the landmark, changing the image of the male and female if appropriate, changing the country name in the text, and the changing the language (Spanish, English, or Dutch) of the posts. Facebook algorithms select which post to show each user.

Re-weighting and external validity

Estimation of weights

For each country, we modeled the probability of being in the nationally representative sample as a function of demographic, time-invariant characteristics such as gender, age and education of the respondent, indicators for the presence of children of 5 years old or younger in the household and elderly (60 years old or older) in the households, as well as the number of household members and the number of children enrolled in school. Finally, we also include region-specific indicators.

We conducted this process country by country. For each country, we used the most recent nationally representative survey available in the Inter-American Development Bank harmonized survey data repository. We estimated the model by fitting a logistic function (logit) and computed predicted probabilities of being in the nationally representative data set ($\hat{p}_{i,c}$) for each respondent i in country c (see Table S2). We then used inverse probability weights ($ipw_{i,c} = 1/(1-\hat{p}_{i,c})$) to, at least in terms of observable characteristics, resemble those from nationally representative surveys.

To prevent differences in response rates from driving the results, we re-scale the within-country weights $ipw_{i,c}$ by the inverse share of re-weighted number of responses per country, relative to the country's population size ($Population_c/(\sum_i^{N_c} ipw_{i,c})$).

Out-of-sample validation

We validate the country-specific weights by looking at their out-of-sample performance. For each country we randomly selected 60% of the observations in the online survey and the household (field) surveys. We used this sample to estimate logit models. We then use the resulting models to predict the probability of being observed in the household survey over the observations in the remaining 40% of the sample, which we call the testing sample. We then re-weight the observations in the testing sample and compare the adjusted means of demographic characteristics to those obtained from household (field) survey data. Table S3 shows this exercise in Columns 3 and 4. We were able to reduce the differences in respondent characteristics between the online and household surveys. This out-of-sample exercise suggests that the improvement in balance is not driven by overfitting. We also show that our online data does not differ substantially from the household survey data in non-targeted moments such as the proportion of respondents by income category.

Countries by type of mobility-restriction policies

The following list details the type of mobility restrictions implemented by each country. Data was collected from official government websites and press articles. The information was collected on April 23rd 2020.

No mandatory policies: Bahamas, Suriname, Trinidad and Tobago, Uruguay. **Curfews:** Dominican Republic, Guyana. **Local Quarantines:** Chile, Jamaica, Mexico. **National Quarantines** Barbados, Bolivia, Colombia, Costa Rica, Ecuador, El Salvador, Panama, Peru.

Datasets

Population and Household Survey Data

Year and Source of Household Survey Data by Country

**Country**	**Survey Name**	**Year**	**Link to source**
Bahamas	LFS	2014	http://www.bahamas.gov.bs/wps/portal/public
Barbados	BSLC	2016	http://sistemasintegrales.cl/project/barbados-survey-of-living-conditions/
Bolivia	ECH	2018	https://www.ine.gob.bo/index.php/herramientas/bases-de-datos-catalogo-anda/bases-de-datos-encuestas-sociales/
Chile	Casen	2017	http://observatorio.ministeriodesarrollosocial.gob.cl/casen-multidimensional/casen/basedatos.php
Colombia	GEIH	2018	http://microdatos.dane.gov.co/index.php/catalog/659/get_microdata
Costa Rica	ENAHO	2018	https://www.inec.cr/noticias/enaho
Dominican Republic	ENCFT	2018	https://www.bancentral.gov.do/a/d/2539
Ecuador	ENEMDU	2018	https://www.ecuadorencifras.gob.ec/estadisticas/
El Salvador	EHPM	2018	http://www.digestyc.gob.sv/index.php/temas/des/ehpm.html
Guyana	LFS	2018	https://statisticsguyana.gov.gy/data/databases/
Jamaica	SLC	2014	https://statinja.gov.jm/living_conditions_poverty.aspx
Mexico	ENIGH	2018	https://www.inegi.org.mx/programas/enh
Panama	EHPM	2018	https://www.inec.gob.pa/publicaciones/Default2.aspx?ID_CATEGORIA=5&ID_SUBCATEGORIA=38
Peru	ENAHO	2018	https://webinei.inei.gob.pe/anda_inei/index.php/catalog/672
Suriname	SLC	2017	https://statistics-suriname.org/en/
Trinidad & Tobago	CSSP	2015	https://cso.gov.tt/methods/classifications/
Uruguay	ECH	2018	http://www.ine.gub.uy/encuesta-continua-de-hogares1

We used the most recent household survey available at IDB's harmonized database repository. Each survey can be accessed on the websites of the specific institutions in each country. The table provides links to original sources for data access. In some occasions, the microdata must be requested directly from the competent body due to privacy restrictions.

Year and Source of Population Data by Country

Country	Year	Link to Source
Bahamas	2010	https://www.bahamas.gov.bs/wps/wcm/connect/22f9b2b0-68fa-4a26-8bd8-474952e42dc2/Population+Projection+Report+2010-2040.pdf?MOD=AJPERES
Barbados	2010	https://web.archive.org/web/20170118220332/http://www.barstats.gov.bb/files/documents/PHC_2010_Census_Volume_1.pdf
Bolivia	2020	https://www.ine.gob.bo/subtemas_cuadros/demografia_html/PC20106.htm
Chile	2020	https://www.ine.cl/estadisticas/sociales/demografia-y-vitales/proyecciones-de-poblacion
Colombia	2020	https://www.dane.gov.co/index.php/estadisticas-por-tema/demografia-y-poblacion/proyecciones-de-poblacion
Costa Rica	2020	https://www.inec.cr/poblacion/estimaciones-y-proyecciones-de-poblacion
Dominican Republic	2020	https://www.one.gob.do/demograficas/proyecciones-de-poblacion
Ecuador	2020	https://sni.gob.ec/proyecciones-y-estudios-demograficos
El Salvador	2020	https://www.transparencia.gob.sv/search?utf8=%E2%9C%93&ft=Proyecciones+municipales
Guyana	2012	https://statisticsguyana.gov.gy/publications/#elementor-tab-content-1465%20%3E%20ul:nth-child(3)%20%3E%20li:nth-child(1)%20%3E%20span
Jamaica	2018	https://statinja.gov.jm/Demo_SocialStats/PopulationStats.aspx
Mexico	2020	https://www.gob.mx/cms/uploads/attachment/file/63977/Documento_Metodologico_Proyecciones_Mexico_2010_2050.pdf
Panama	2020	https://www.inec.gob.pa/publicaciones/Default3.aspx?ID_PUBLICACION=499&ID_CATEGORIA=3&ID_SUBCATEGORIA=10
Peru	2020	http://proyectos.inei.gob.pe/web/biblioineipub/bancopub/Est/Lib0846/libro.pdf
Suriname	2012	https://statistics-suriname.org/en/census-statistics-2012/
Trinidad and Tobago	2011	https://cso.gov.tt/census/2011-census-data/
Uruguay	2020	http://www.ine.gub.uy/estimaciones-y-proyecciones

The projected population data for Panama excludes Comarca Guna de Madugandi and Comarca Guna de Wargandi. Population data for years 2018 through 2020 is projected.

Statistical Analysis Details

Figure 1

Fig 1 shows how job loss and business closures vary throughout the income distribution. Job loss is a dummy variable that equals one if the respondent indicates that they lost their job within the last week, two weeks or month (these horizons were randomly assigned). Similarly, the variable for business closures equals one if subjects report that their business was forced to close by the government or by lack of demand within the same randomly assigned time horizon.

To explore differences in job loss and business closure across the income distribution, responses are first aggregated at the country and income bracket level using within-country weights as described in the Supplementary Information Appendix. Country averaged outcomes are then regressed by OLS on indicators for income bin and control for country fixed effects, weighting by country population. Because there are only have 17 countries in the sample, using standard clustered standard errors would result in incorrect inference. Therefore, the analysis follow [1] by aggregating the data at the country level by income category. Fig 1 shows the point estimates and 95 percent confidence intervals for each income bin average. As robustness, results are shown for the raw pooled data, without applying weights in Figure S2.

Figure 2

Fig 2 presents a cross-country comparison of the shares of households who lost their livelihoods as a function of the share of self-employed workers in the economy. For this, respondents that reported that at least one household member lost their job during or had to close their businesses during the survey reference period were identified, and calculated shares using weights to correct for sampling issues (See Supplementary Information section). Data corresponding to the share of self-employed workers was obtained from the World Bank's World Development Indicators.

Figure 3

The share of household income in each income bin for incomes reported by respondents corresponding to January 2020 and April 2020 are presented in Fig 3. The sample is restricted to responses collected between April 13th to May 1st 2020 to include all countries. Shares are calculated by re-weighting counts by within- and across-country weights as detailed in Supplementary Material section Estimation of weights. As robustness, results are shown for the raw pooled data, without applying weights in Figure S3.

Figure 4

Fig 4 shows the share of respondents supporting extending the lockdown policies by at least one month over time since the first COVID-19 case in the country. One challenge to explore how support changes over time is that the timing of responses varies between countries. Over time as new countries enter the sample, the change in sample composition could bias our results. We address this issue in two steps. First, we construct new weights and re-weight respondents after day 31 to match those responding at the beginning of the series (days 30 and 31) on observable demographic characteristics.

A model was estimated by fitting a logistic function (logit) and computed predicted probabilities of having responded to the survey on day 30 or 31 relative to first case.

Explanatory variables included were: demographic characteristics such as household size, age indicators for: presence of children or elderly, having felt hunger, woman, education primary or less, college education or higher, household income categories (<1, 1-2,2-3, 8-11,>11), and for day of week. Then, inverse probability weights were estimated based on the propensity score obtained.

Second, OLS regressions were separately estimated for respondents whose household member lost job or closed business and for those who did not, where the dependent variable is an indicator variable that equals one if they support extending the lockdown by at least a month, and zero otherwise. The independent variables are indicators for days since first COVID-19 case, day of week indicators, and country fixed effects. The regression is weighted by the inverse probability weight described in step one and employs robust standard errors. Fig 4 presents the point estimates and 95 percent confidence intervals for these estimates. Similar estimates when using the pooled raw data without re-weighting are presented in Figure S4.

Table 1

Table **??** reports comparisons of differences in the relevant outcomes between respondents whose households experienced a loss of livelihood during the pandemic period and those that did not. To control for time-varying characteristics, we perform comparisons focusing on respondents in the same locality and who completed the survey during the same day. We exploit granular data from over 3000 localities in 17 countries. Thus, we focus our analysis on the subset of 18,000 locality-date-of-response cells that include more than one observation. This approach allows us to isolate time-varying locality shocks, and thus purge regional confounding factors. In addition we control for industry sector fixed effects, to prevent differences in the sectors related to the household's main economic activities from driving the results, as exposure to the effects of the pandemic may vary across sectors.

We operationalize our approach by estimating the following specification, which is similar to that used in reference [2]:

$$\mathrm{Y_{i,l,c,t}} = \beta \mathrm{Lostlivelihood_{i,l,c,t}} + \mathbf{X}_{i,c,l,t}\Sigma + \delta_{l,c,t} + \theta_s + \epsilon_{i,l,c,t} \tag{1}$$

Here, $Y_{i,l,c,t}$ denotes the outcome of interest corresponding to respondent i in locality l from country c collected in date t. Lost livelihood$_{i,l,c,t}$ is an indicator of whether any member of the respondent's household lost her/his job or closed her business during the the past week, two-weeks, or month. $\mathbf{X}_{i,c,l,t}$ is vector of demographic characteristics of the respondent (age, education level and gender) and of household characteristics (household size, presence of children younger than 5, presence of school-age children, and people 60 years old or older). $\delta_{l,c,t}$ denotes locality-date fixed effects, and θ_s denotes industry fixed effects based on the main pre-pandemic source of income of the respondent's household. $\epsilon_{i,c,l,t}$ denotes unobserved shocks. To account for possible serial correlation of outcomes within localities, we cluster the standard errors at the locality-country level.

The parameter of interest, β, is reported in Table 1 and captures within-locality differences in outcomes between respondents whose households experienced a loss of livelihood during the pandemic and those who didn't. The reported models are estimated using weights to achieve country-level representativeness, to correct for differences in sample size across countries, and to provide higher based on country population size. All results are robust to excluding these weights (See Robustness Section in Supplementary Material).

Dependent variables: *Went hungry* is an indicator of whether any household member went hungry during the past week due to lack of food. *Eats less healthy* takes

the value of one if the respondent somewhat or totally agrees with the statement "I eat more unhealthy foods than normal". *Gifts/Loans* is an indicator of whether the respondent's household received a gift or transfer from either friends or relatives during the preceding week. Gov. Priority is an indicator of whether the respondent somewhat agrees or totally agrees with the statement "The government's priority should be to stop the spread of the pandemic". *Lockdown (>= month)* is an indicator of whether the respondent reports agreeing with closing non-essential business for one month or longer. As this question was asked only to people that reported agreeing with policies that require non-essential businesses to close, *Lockdown*(>= *month*) takes the value of zero when the respondent reported not supporting measures of keeping non-essential businesses closed at all, regardless of the time.

Figure 2

To investigate whether the correlates of job loss or business closures and household outcomes are stronger in countries with high levels of informality, we also estimate the following specification:

$$\begin{aligned} \mathrm{Y_{i,l,c,t}} = \quad & \beta_1 \mathrm{Lostlivelihood_{i,l,c,t}} + \beta_2 \mathrm{Lostlivelihood_{i,l,c,t}} \times \mathrm{Self-employment}_c \\ & + \mathbf{X}_{i,c,l,t}\Sigma + \delta_{l,c,t} + \theta_s + \epsilon_{i,l,c,t} \end{aligned} \quad (2)$$

Where *Self-employment* denotes the share of self-employed workers in country c and was obtained from the World Bank's World Development Indicators, using the most-recent observations for each country. We also report results using the share of informal workers (as a share of non-agricultural workers) in each country. This information was not available for Suriname, Jamaica, Trinidad and Tobago, Barbados, and Bahamas. See results in Table S4.

Supplementary Figures

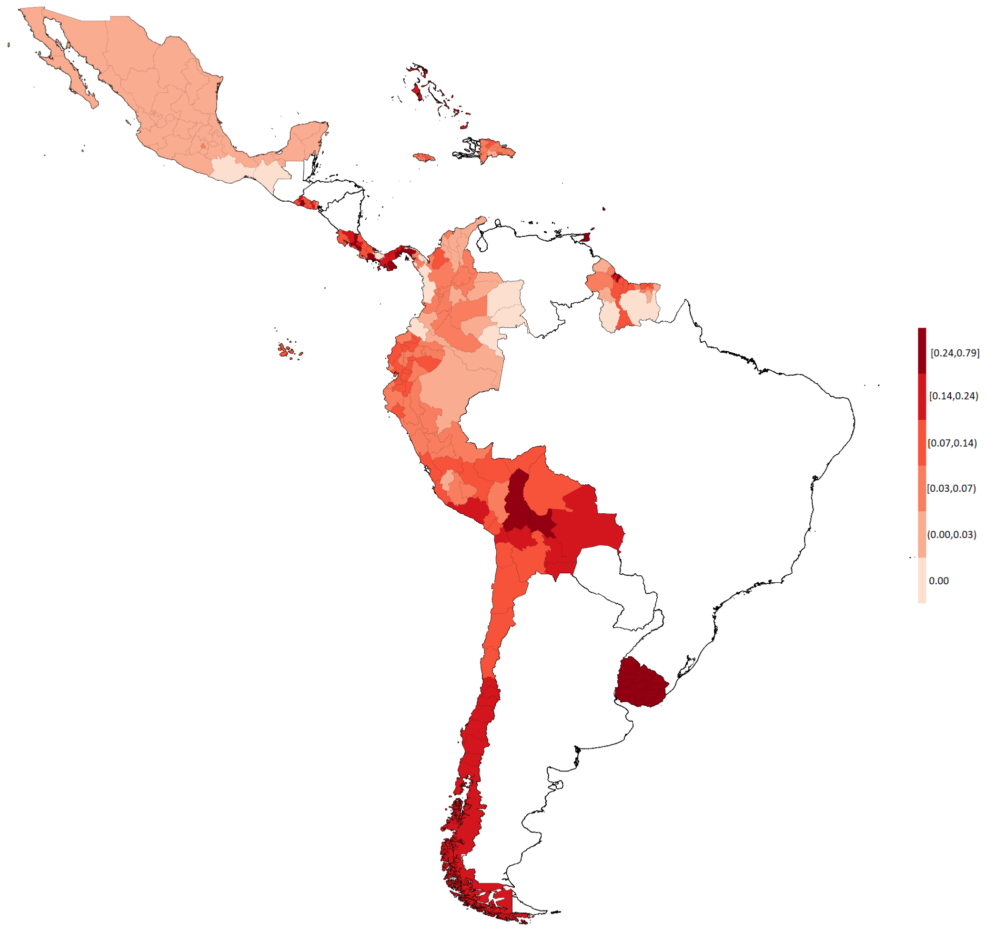

Fig S1. Sample has broad geographic coverage at the sub-national level. The sub-national regions of each country in the sample are shaded according to number of observations as a share of population (in %). Sources of population data for each country are shown in the Supplementary Information section. Administrative boundary shapefiles were obtained from the GADM database (www.gadm.org).

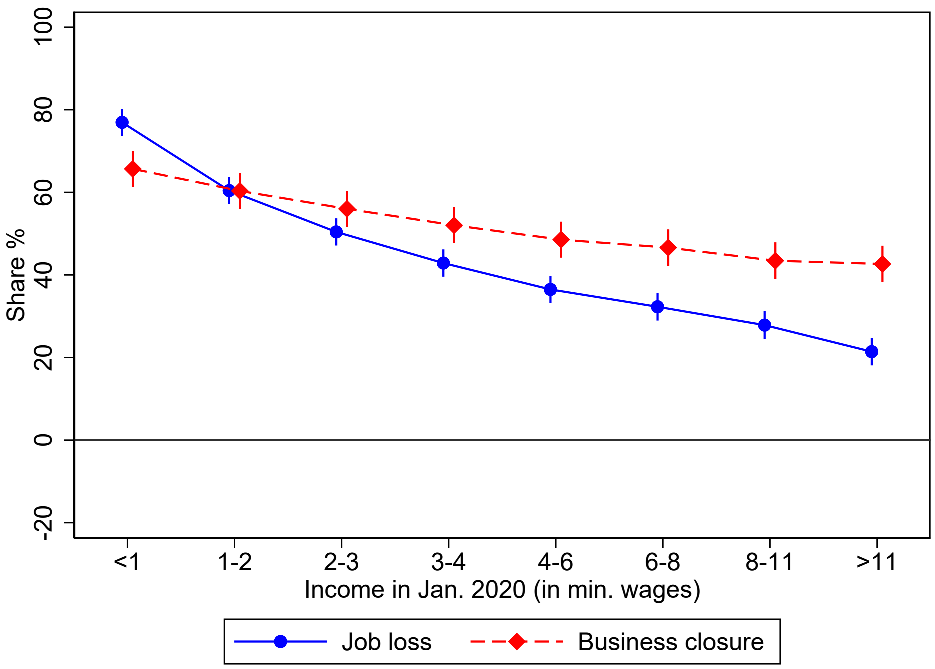

Fig S2. Higher rates of job loss and business closure among households in the lowest income group. Point estimates and 95% confidence intervals from a regression of the outcome on income bin indicators and country fixed effects. Pooled data (no weights), robust standard errors. See the Empirical Methods section in the Supplementary Information section for more details.

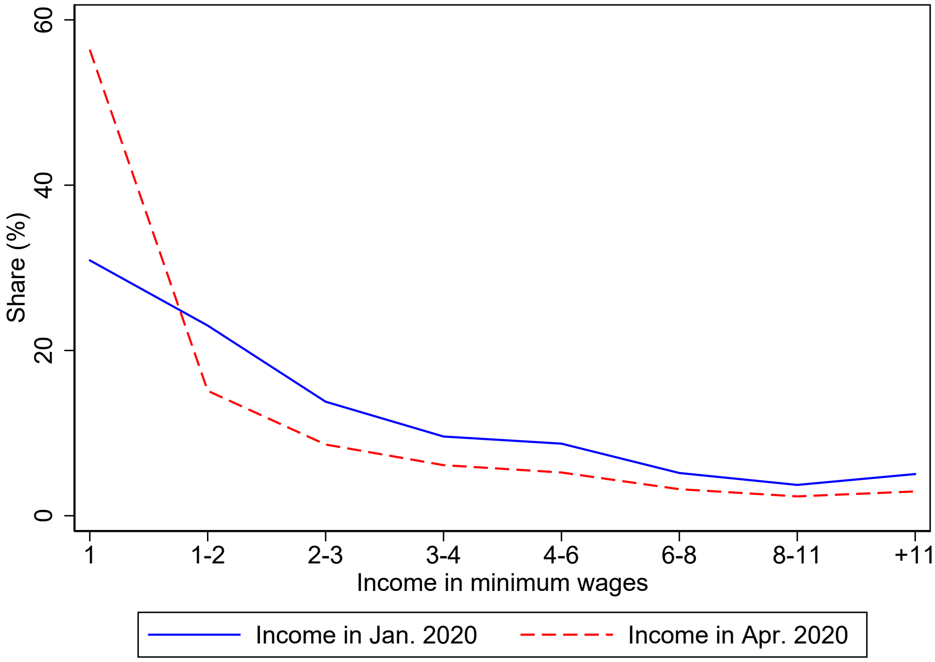

Fig S3. The share of households in the bottom part of the income distribution is expected to increase. Shares of households in each income bin for incomes reported for January 2020 and April 2020. Pooled data (no weights). See the Empirical Methods section in the Supplementary Information section for more details.

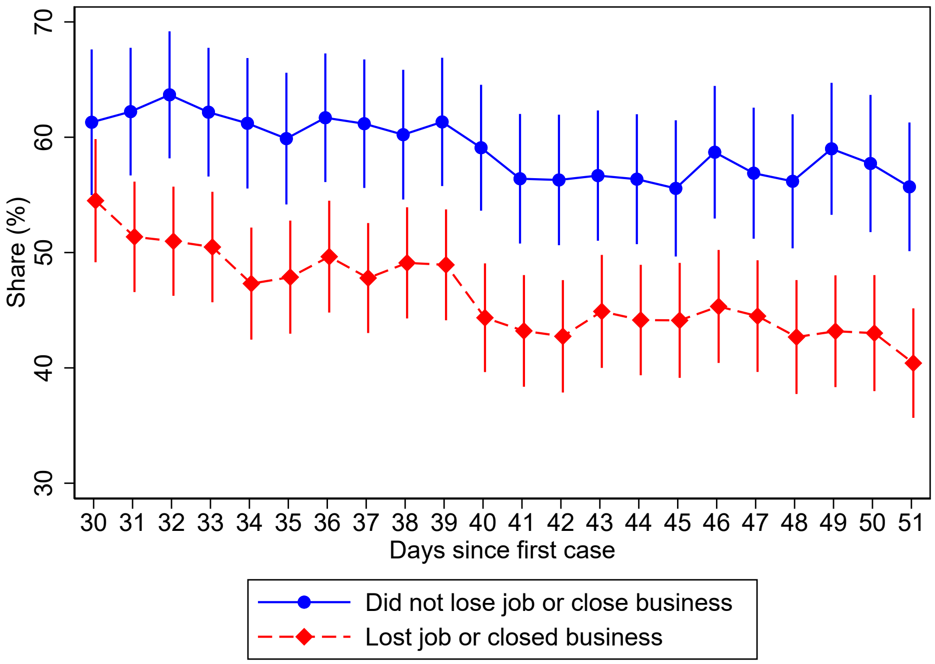

Fig S4. Support for extending lockdown policies declines more among households thatlost their livelihoods. Point estimates and 95% confidence intervals for the share of respondents supporting extending lockdown policies according to days since first COVID-19 case in the country. Pooled data (no weights). See the Empirical Methods section in the Supplementary Information section for more details.

Supplementary Tables

Table S1. Date of Launch and Number of Observations by Country

Country	(1) Launch Date	(2) No. of Observations	(3) % of Localities with Observations
Chile	3/27/2020	35,556	97%
Bolivia	4/1/2020	25,970	83%
Panama	4/3/2020	15,521	100%
Uruguay	4/3/2020	21,191	64%
Peru	4/7/2020	25,452	47%
Mexico	4/13/2020	19,483	51%
Costa Rica	4/13/2020	9,151	90%
Colombia	4/15/2020	23,458	71%
El Salvador	4/16/2020	13,456	50%
Dominican Republic	4/16/2020	7,965	94%
Barbados	4/16/2020	2,072	100%
Jamaica	4/16/2020	2,547	91%
Guyana	4/16/2020	1,670	96%
Bahamas	4/16/2020	896	81%
Trinidad and Tobago	4/16/2020	4,683	100%
Ecuador	4/16/2020	18,688	68%
Suriname	4/17/2020	565	100%

Date of launch is the date on which the social media posts began. The date that the survey was rolled out in each country was largely determined by bureaucratic processes and approvals. With the except of Costa Rica, data collection in each country continued until April 30, 2020. The number of observations for each country reflected the number in the sample after data cleaning. The percent of localities is the percent of localities of each country for which we have one or more observations in the sample.

Table S2. Parameters of logit models for the probability of being in the nationally representative survey

	Bahamas	Barbados	Bolivia	Chile	Colombia	Costa Rica	Dominican Rep.	Ecuador	El Salvador	Guyana	Jamaica	Mexico	Peru	Panama	Suriname	Trinidad & Tobago	Uruguay
Household size	-0.002	-0.575***	-0.206***		-0.123***	-0.073***	-0.061***	-0.084***	-0.047***	-0.195***	-0.326***	-0.048***	-0.085***	-0.097***	-0.197***	-0.067***	-0.125***
	(0.034)	(0.020)	(0.008)		(0.007)	(0.013)	(0.020)	(0.009)	(0.012)	(0.031)	(0.044)	(0.008)	(0.006)	(0.011)	(0.032)	(0.009)	(0.009)
# of children in school age	-0.814***		0.389***	0.225***	0.168***	0.124	0.138**	0.563***	0.153*	0.973***	2.720***	0.583***	0.967***	0.080			0.665***
	(0.135)		(0.033)	(0.008)	(0.040)	(0.096)	(0.066)	(0.027)	(0.089)	(0.108)	(0.201)	(0.039)	(0.033)	(0.059)			(0.051)
Children under 5 years old in household (%)	-0.238**		-0.455***		-0.348***	-0.264***	-0.257***	-0.835***	-0.299***	-0.891***	-3.600***	-0.689***	-1.104***	-0.097*			-0.701***
	(0.112)		(0.030)		(0.037)	(0.093)	(0.061)	(0.023)	(0.087)	(0.082)	(0.203)	(0.035)	(0.030)	(0.056)			(0.047)
Elderly in household (%)	-0.520***	0.702***	-1.010***	0.038**	-0.167***	0.073*	-0.329***	-0.072**	-0.183***	-0.013	0.157	-0.103***	-0.280***	0.028	0.138	0.066	0.183***
	(0.133)	(0.066)	(0.030)	(0.016)	(0.024)	(0.040)	(0.058)	(0.032)	(0.037)	(0.110)	(0.155)	(0.025)	(0.022)	(0.039)	(0.177)	(0.041)	(0.026)
# of children enrolled in school	-0.831***	-1.060***	-0.325***	-1.366***	-0.737***	-0.699***	-0.460***	-0.360***	-0.234***	-0.964***	-1.879***	-0.564***	-0.645***	-0.338***	-0.588***	-1.100***	-0.983***
	(0.122)	(0.065)	(0.027)	(0.016)	(0.021)	(0.033)	(0.048)	(0.028)	(0.032)	(0.100)	(0.168)	(0.021)	(0.019)	(0.034)	(0.166)	(0.040)	(0.022)
Respondent is a female	-0.071	0.000	-1.108***	-2.543***	-2.018***	-2.227***	-0.996***	-3.228***	-2.272***	-1.208**	1.027*	-1.843***	-1.773***	-2.076***	-1.642		-1.671***
	(1.122)	(.)	(0.186)	(0.127)	(0.182)	(0.197)	(0.264)	(0.149)	(0.143)	(0.532)	(0.580)	(0.167)	(0.192)	(0.587)	(1.061)		(0.191)
Completed Primary	-2.205**	0.110	-3.632***	-4.496***	-3.329***	-3.297***	-3.660***	-3.478***	-3.874***	-4.066***	-1.585***	-5.006***	-4.477***	-4.905***	-5.261***		-4.638***
	(1.029)	(0.349)	(0.167)	(0.126)	(0.178)	(0.197)	(0.218)	(0.145)	(0.141)	(0.505)	(0.545)	(0.163)	(0.180)	(0.580)	(1.018)		(0.190)
Completed Secondary	-5.185***	-1.623***	-6.316***	-6.035***	-5.904***	-5.698***	-6.427***	-6.900***	-6.614***	-6.726***	0.000	-6.797***	-6.041***	-8.058***	-10.927***		-5.377***
	(1.021)	(0.346)	(0.165)	(0.125)	(0.176)	(0.196)	(0.217)	(0.143)	(0.139)	(0.508)	(.)	(0.162)	(0.180)	(0.580)	(1.070)		(0.190)
University/Vocational Training or Higher	0.010**	-0.010***	0.039***	-0.017***	0.004***	0.011***	0.016***	0.007***	0.006***	0.009***	0.018***	-0.017***	0.015***	0.019***	-0.003	0.017***	0.002***
	(0.004)	(0.002)	(0.001)	(0.001)	(0.001)	(0.001)	(0.002)	(0.001)	(0.001)	(0.003)	(0.005)	(0.001)	(0.001)	(0.001)	(0.006)	(0.001)	(0.001)
Age of respondent			-0.385***		-0.181***	-0.398***	-0.452***	-0.611***	-0.500***	0.044	0.214	-0.221***	-0.282***	-0.082*		-0.011	-0.065**
			(0.032)		(0.026)	(0.045)	(0.055)	(0.034)	(0.040)	(0.113)	(0.151)	(0.027)	(0.023)	(0.044)		(0.048)	(0.029)
N	6413	11314	48150	201380	159692	34367	19722	58403	61294	10323	13363	199884	115388	41597	5376	29008	103015

$*p < 0.1, **p < 0.05, ***p < 0.01$

The table presents estimates of coefficients from a logit model of the probability of being the nationally representative household survey as a function of demographic characteristics. The models were estimated separately for each country. All models also included region-specific indicators, except in the case of Ecuador in which data regarding regions was not available. Empty cells in the table imply that the relevant variable was not available in the household surveys. Robust standard errors are presented in parenthesis.

Table S3. Differences between Online Survey Data and Household (field) Survey Data

	Full Sample		Testing sample	
	(1)	(2)	(3)	(4)
	Online	HH Survey	Online	HH Survey
	(Raw)	(Sampling weights)	(Re-weighted)	(Sampling weights)
Household size	4.27	3.87	4.30	3.87
Children under 5 years old in household (%)	0.28	0.24	0.33	0.24
Elderly in household (%)	0.35	0.36	0.36	0.36
# of children enrolled in school	1.04	0.81	0.89	0.81
Respondent is a female	0.70	0.52	0.58	0.52
Completed Primary	0.03	0.36	0.31	0.36
Completed Secondary	0.22	0.32	0.30	0.32
University/Vocational Training or Higher	0.74	0.12	0.24	0.12
Age of respondent	38.13	43.07	39.62	43.04
Income categories				
0-0.5 MW	0.10	0.14	0.17	0.14
0.5-1 MW	0.16	0.09	0.22	0.09
1-2 MW	0.21	0.20	0.22	0.20
2-3 MW	0.14	0.15	0.11	0.16
3-4 MW	0.11	0.10	0.08	0.10
4-6 MW	0.10	0.12	0.07	0.12
6-8 MW	0.06	0.06	0.04	0.06
8-11 MW	0.05	0.04	0.02	0.04
11+ MW	0.07	0.09	0.06	0.09
Countries	17	17	17	17
Observations	230,540	913,694	92,715	364,972

The table presents means of household and survey respondent demographic characteristics using data from the online survey and nationally representative surveys, and pooling observations from all study countries (weighting by country size). Column (1) reports raw means using all the observations from the online surveys. Column (2) reports means using all available observations in the household (field) surveys using sampling weights. Column (3) reports means from the online survey data using only data from the testing sample (i.e., the sample not used for the estimation of the inverse probability weights). Column (4) reports means using data from the household (field) surveys corresponding to the testing sample. The testing sample corresponds to a randomly selected subsample corresponding to 40% of all the observations in the online and household (field) surveys. MW stands for national minimum wage. The inverse probability weights are computed based on logit models of the probability of being observed in the household survey which are estimated country by country. The models include including age, gender, and education categories of the respondent as well as household-level demographic characteristics such as the presence of children younger than 5 years old, the presence of elderly children in the household, # of children enrolled in school, household size, as well as region fixed effects.

Table S4. Loss of livelihoods and changes in well-being, and policy support (unweighted results)

Panel A: Locality X date Fixed effects - unweighted						
	(1)	(2)	(3)	(4)	(5)	(6)
	Decreased income	Went hungry	Eats less healthy	Gift/Loan	Gov. Priority	Lockdown (>= *month*)
Lost job or closed business	0.293***	0.096***	0.073***	0.213***	-0.026***	-0.030***
	(0.007)	(0.003)	(0.003)	(0.003)	(0.002)	(0.003)
Observations	188152	200594	176198	200421	198410	125359
Adjusted R2	0.235	0.140	0.036	0.157	0.035	0.430
Panel B: Country X date fixed effects (weighted)						
	(1)	(2)	(3)	(4)	(5)	(6)
	Decreased income	Went hungry	Eats less healthy	Gift/Loan	Gov. Priority	Lockdown (>= *month*)
Lost job or closed business	0.245***	0.162***	0.069***	0.216***	-0.036***	-0.008
	(0.021)	(0.025)	(0.016)	(0.018)	(0.008)	(0.018)
Observations	202832	215410	190065	215236	213188	139800
Adjusted R2	0.266	0.244	0.097	0.192	0.110	0.111
Panel C: Country X date fixed effects (unweighted)						
	(1)	(2)	(3)	(4)	(5)	(6)
	Decreased income	Went hungry	Eats less healthy	Gift/Loan	Gov. Priority	Lockdown (>= *month*)
Lost job or closed business	0.291***	0.100***	0.074***	0.212***	-0.026***	-0.031***
	(0.023)	(0.010)	(0.005)	(0.009)	(0.003)	(0.008)
Observations	204464	217249	191801	217075	214980	141617
Adjusted R2	0.236	0.124	0.031	0.149	0.026	0.026

$*p < 0.1, **p < 0.05, ***p < 0.01$

Panel A reports unweighted regression coefficients capturing the relationship between livelihood losses during the pandemic and outcomes. Each column reports results of a regression of the dependent variable on an indicator of whether any household member either lost a job or closed a business and a vector of covariates. In addition, all regressions control for locality × day of survey completion fixed effects (18,764), as well as economic-sector fixed effects. Standard errors are clustered at the locality level (3,165). Panels B and C replicate the results using country-date fixed effects (300) and standard errors clustered at the country level (17). See the Empirical Methods section in the main text for more details.

Table S5. Impacts of livelihood loss on income and food security by labor market characteristics

**Panel A: Impacts on food security by country-level rates of self-employment**			
	(1)	(2)	(3)
	Decreased income	Went hungry	Eats less healthy
Lost job or closed business × % Self-Employed	-0.006***	0.002***	0.000
	(0.001)	(0.000)	(0.000)
Lost job or closed business	0.488***	0.019	0.066***
	(0.022)	(0.020)	(0.022)
Observations	186521	198726	174452
Adjusted R-squared	0.437	0.545	0.350

**Panel B: Impacts on food security by country-level rates of informality**			
	(1)	(2)	(3)
	Decreased income	Went hungry	Eats less healthy
Lost job or closed business × % Informal workers	-0.003***	0.002***	0.001**
	(0.000)	(0.000)	(0.000)
Lost job or closed business	0.421***	0.017	0.038**
	(0.013)	(0.015)	(0.016)
Observations	179040	190453	166581
Adjusted R-squared	0.416	0.548	0.355

$*p < 0.1, **p < 0.05, ***p < 0.01$

Panel A reports regression coefficients capturing the relationship between livelihood losses during the pandemic and outcomes, as a function of the share of self-employed workers in each country. Each column reports results of a regression of the dependent variable on an indicator of whether any household member either lost a job or closed a business, and an interaction term of job loss or business closure with the share of self-employed workers in each country, and a vector of covariates. In addition, all regressions control for locality × day of survey completion fixed effects (18,764), as well as economic-sector fixed effects. Standard errors are clustered at the locality level (3,165). Panels B replicates the results using the percentage of informal workers as in each country instead of the share of self-employed workers. See the Empirical Methods section in the main text for more details.

References not included in main article

1. Bertrand M, Duflo E, Mullainathan S. How Much Should We Trust Differences-In-Differences Estimates?. The Quarterly Journal of Economics. 2004 02;119(1):249–275.

2. Gertler P, Gruber J. Insuring Consumption Against Illness. American Economic Review. 2002 March;92(1):51–70.
