# Supplementary material for: The unequal impact of the coronavirus pandemic: Evidence from seventeen developing countries
Source: PLoS One. 2020 Oct 7;15(10):e0239797. doi: 10.1371/journal.pone.0239797 (PMC7540865; doi:10.1371/journal.pone.0239797)
Supplement: Attachment — Submitted filename: PONE-D-20-15019_reviewer_RB.pdf [file pone.0239797.s002.pdf]

PLOS ONE

The unequal impacts of the Coronavirus pandemic: Evidence from seventeen developing countries

--Manuscript Draft--

**Manuscript Number:**	PONE-D-20-15019
**Article Type:**	Research Article
**Full Title:**	The Uunequal Iimpacts of the Coronavirus Ppandemic: Evidence from Sseventeen Ddeveloping Ccountries
**Short Title:**	The Uunequal Iimpacts of the Coronavirus Ppandemic
**Corresponding Author:**	Nicolas Bottan<br>Cornell University College of Human Ecology<br>Ithaca, UNITED STATES
**Keywords:**	Coronavirus; Inequality; Labor Market; Household Survey
**Abstract:**	The current coronavirus pandemic is an unprecedented public health challenge that has devastating economic impacts for households. Using a sample of 230,540 respondents to online surveys in 17 countries in Latin America and the Caribbean, we show that the economic impacts are large and unequal: 45% percent of respondents report that a household member lost a job, and among households owning small businesses, 59% percent of respondents report that a household member closed their business. Among households with lowest income prior to the pandemic, 71% percent report that a household member lost their job and 61% percent report that a household member closed their business. Declines in food security and healthiness are among the disproportionate impacts. Our results provide evidence that the current public health crisis will exacerbate economic inequality, and are among the first estimates of the labor-market and well-being impacts of the pandemic in developing countries.
**Order of Authors:**	Nicolas Bottan
	Bridget Hoffmann
	Diego Vera-Cossio
**Additional Information:**	
**Question**	**Response**
**Financial Disclosure**<br><br>Enter a financial disclosure statement that describes the sources of funding for the work included in this submission. Review the submission guidelines for detailed requirements. View published research articles from *PLOS ONE* for specific examples.<br><br>This statement is required for submission and **will appear in the published article** if the submission is accepted. Please make sure it is accurate.	Bridget Hoffmann and Diego Vera-Cossio were joint recipients of the Inter-American Development Bank's Coronavirus research funds (RG-E1700) - www.iadb.org.<br>The funders had no role in study design, data collection and analysis, decision to publish, or preparation of the manuscript.

Powered by Editorial Manager® and ProduXion Manager® from Aries Systems Corporation

**Unfunded studies**<br>Enter: *The author(s) received no specific funding for this work.*<br><br>**Funded studies**<br>Enter a statement with the following details:<br>• Initials of the authors who received each award<br>• Grant numbers awarded to each author<br>• The full name of each funder<br>• URL of each funder website<br>• Did the sponsors or funders play any role in the study design, data collection and analysis, decision to publish, or preparation of the manuscript?<br>• **NO** - Include this sentence at the end of your statement: *The funders had no role in study design, data collection and analysis, decision to publish, or preparation of the manuscript.*<br>• **YES** - Specify the role(s) played.<br><br>* typeset	
**Competing Interests**<br><br>Use the instructions below to enter a competing interest statement for this submission. On behalf of all authors, disclose any competing interests that could be perceived to bias this work—acknowledging all financial support and any other relevant financial or non-financial competing interests.<br><br>This statement **will appear in the published article** if the submission is accepted. Please make sure it is accurate. View published research articles from *PLOS ONE* for specific examples.	The authors have declared that no competing interests exist.

Powered by Editorial Manager® and ProduXion Manager® from Aries Systems Corporation

**NO authors have competing interests**<br><br>Enter: *The authors have declared that no competing interests exist.*<br><br>**Authors with competing interests**<br><br>Enter competing interest details beginning with this statement:<br><br>*I have read the journal's policy and the authors of this manuscript have the following competing interests: [insert competing interests here]*<br><br>* typeset	
**Ethics Statement**<br><br>Enter an ethics statement for this submission. This statement is required if the study involved:<br><br>• Human participants<br>• Human specimens or tissue<br>• Vertebrate animals or cephalopods<br>• Vertebrate embryos or tissues<br>• Field research<br><br>Write "N/A" if the submission does not require an ethics statement.<br><br>General guidance is provided below. Consult the submission guidelines for detailed instructions. **Make sure that all information entered here is included in the Methods section of the manuscript.**	This study involving human subjects has been reviewed and approved by the IRB at Cornell University (#2003009501).<br>Informed consent was obtained on initial page of online survey.

Powered by Editorial Manager® and ProduXion Manager® from Aries Systems Corporation

**Format for specific study types**<br><br>**Human Subject Research (involving human participants and/or tissue)**<br>• Give the name of the institutional review board or ethics committee that approved the study<br>• Include the approval number and/or a statement indicating approval of this research<br>• Indicate the form of consent obtained (written/oral) or the reason that consent was not obtained (e.g. the data were analyzed anonymously)<br><br>**Animal Research (involving vertebrate animals, embryos or tissues)**<br>• Provide the name of the Institutional Animal Care and Use Committee (IACUC) or other relevant ethics board that reviewed the study protocol, and indicate whether they approved this research or granted a formal waiver of ethical approval<br>• Include an approval number if one was obtained<br>• If the study involved *non-human primates*, add *additional details* about animal welfare and steps taken to ameliorate suffering<br>• If anesthesia, euthanasia, or any kind of animal sacrifice is part of the study, include briefly which substances and/or methods were applied<br><br>**Field Research**<br><br>Include the following details if this study involves the collection of plant, animal, or other materials from a natural setting:<br>• Field permit number<br>• Name of the institution or relevant body that granted permission	
**Data Availability**<br><br>Authors are required to make all data underlying the findings described fully available, without restriction, and from the time of publication. PLOS allows rare exceptions to address legal and ethical concerns. See the PLOS Data Policy and FAQ for detailed information.	Yes - all data are fully available without restriction

Powered by Editorial Manager® and ProduXion Manager® from Aries Systems Corporation

A Data Availability Statement describing where the data can be found is required at submission. Your answers to this question constitute the Data Availability Statement and **will be published in the article**, if accepted.<br><br>**Important:** Stating 'data available on request from the author' is not sufficient. If your data are only available upon request, select 'No' for the first question and explain your exceptional situation in the text box.<br><br>Do the authors confirm that all data underlying the findings described in their manuscript are fully available without restriction?	
**Describe where the data may be found in full sentences. If you are copying our sample text, replace any instances of XXX with the appropriate details.**<br><br>• If the data are **held or will be held in a public repository**, include URLs, accession numbers or DOIs. If this information will only be available after acceptance, indicate this by ticking the box below. For example: *All XXX files are available from the XXX database (accession number(s) XXX, XXX.).*<br>• If the data are all contained **within the manuscript and/or Supporting Information files**, enter the following: *All relevant data are within the manuscript and its Supporting Information files.*<br>• If neither of these applies but you are able to provide **details of access elsewhere**, with or without limitations, please do so. For example:<br><br>*Data cannot be shared publicly because of [XXX]. Data are available from the XXX Institutional Data Access / Ethics Committee (contact via XXX) for researchers who meet the criteria for access to confidential data.*<br><br>*The data underlying the results presented in the study are available from (include the name of the third party*	Data and replication files are currently openly available at:<br>https://www.nicolasbottan.com/InequalityCoronavirus.html<br>Data will be moved to a public repository/DOI after acceptance

Powered by Editorial Manager® and ProduXion Manager® from Aries Systems Corporation

and contact information or URL).<br>• This text is appropriate if the data are owned by a third party and authors do not have permission to share the data.<br><br>* typeset	
Additional data availability information:	Tick here if the URLs/accession numbers/DOIs will be available only after acceptance of the manuscript for publication so that we can ensure their inclusion before publication.

Powered by Editorial Manager® and ProduXion Manager® from Aries Systems Corporation

Click here to download Manuscript PLOS_ONE_Covid19.pdf

The unequal impact of the coronavirus pandemic: Evidence from seventeen developing countries

Nicolas Bottan[1%]*, Bridget Hoffmann[2%], Diego Vera-Cossio[2%],

1 Policy Analysis and Management, Cornell University, Ithaca, NY 14853, USA.
2 Research Department, Inter-American Development Bank, Washington, DC 20577, USA.

%These authors contributed equally to this work.
(Membership list can be found in the Acknowledgments section.
* nicolas.bottan@cornell.edu

Abstract

The current coronavirus pandemic is an unprecedented public health challenge that has devastating economic impacts for households. Using a sample of 230,540 respondents to online surveys ~~in 17 countries in~~ among 17 countries of Latin America and the Caribbean, we show that the economic impacts are large and unequal: 45~~%~~ percent of respondents report that a household member lost a job, and among households owning small businesses, 59~~%~~ percent of respondents report that a household member closed their business. Among households with lowest income prior to the pandemic, 71~~%~~ percent report that a household member lost their job and 61~~%~~ percent report that a household member closed their business. Declines in food security and healthiness are among the disproportionate impacts. ~~Our results~~ The study findings provide evidence that the current public health crisis will exacerbate economic inequality, and are among the first estimates of the labor-market and well-being impacts of the pandemic in developing countries.

Author summary

Using a household survey of 230,540 respondents in seventeen developing countries, we document the devastating labor market impacts of the coronavirus pandemic. We find that the relationship between job loss and business closure is monotonically decreasing with income prior to the pandemic, exacerbating existing inequality. These unequal labor market impacts lead to unequal impacts beyond income, including food security and nutrition. Cross-country comparisons suggest that the impacts of the pandemic are stronger in countries with higher rates of informality. Not only are households from high-informality countries losing their livelihoods at higher rates, but they are also less resilient to these negative shocks. We provide some evidence suggesting that differences in the strength of lockdown policies across countries and the ability to work from home can be related to these impacts. Furthermore, ~~labor market impacts~~ the impacts of labor market affect policy preferences, as support for policies to contain the coronavirus is weaker among those experiencing job loss or business closure. Our results are among the first and only estimates of the economic impacts of the coronavirus pandemic on households outside of developed nations and we find impacts that are larger than those reported in developed countries.

Introduction

Several prominent economists have posited that inequality is the principal economic issue of our era [1–4]. Recent economic slowdowns, such as the Great Recession of 2008-2009, increased economic inequality [5–7]. However, compared to other economic downturns, the COVID-19 pandemic alters economic activity through different channels and on an accelerated timeline.

To slow the spread of COVID-19, governments have implemented regulations that require social distancing, close of non-essential businesses, restrict travel, and in many cases require residents to remain in their homes [8]. The human interactions that drive the economy, such as working together in enclosed areas and enjoying entertainment activities, have been discouraged, restricted~~,~~ or banned. Residents are complying with these measures, report strong support for these regulations [9–11], and actively seek for information [12]. Although these measures are necessary for public health, recent evidence from developed countries suggests that they have negative economic impacts in the short-run [13, 14], and ~~that~~ they can potentially deepen the pre-existing gaps [15]. These impacts could be exacerbated in developing countries with more vulnerable workers and firms, as well as lower levels of state capacity to face the pandemic.

Using a large-scale online household survey, ~~we show~~ the study shows that the COVID-19 pandemic has unequal economic impacts on households in Latin America and the Caribbean that will further exacerbate inequality. Research on economic inequality is particularly relevant in Latin America and the Caribbean. Although inequality and poverty declined over the past decade [16, 17], prior to the pandemic, the region still had the highest income inequality in the world [18], and a large share of citizens were vulnerable to falling back into poverty due to economic shocks [19].

Data collection and validation

We conducted online surveys of households in 8 South American countries (Chile, Colombia, Bolivia, Ecuador, Guyana, Peru, Suriname, and Uruguay), 4 North and Central American countries (Costa Rica, El Salvador, Mexico, and Panama), and 5 Caribbean countries (Dominican Republic, Bahamas, Barbados, Jamaica, and Trinidad and Tobago), resulting in a sample of 230,540 observations. The survey was first launched on March 27, 2020 ~~and was launched in all~~ among all the countries in our sample by April 17, 2020. With the exception of Costa Rica, data collection continued until April 30, 2020. Therefore, the majority of the data were collected during the last two weeks of April 2020. The number of observations by country ranges from 565 to 35,556 (see Table S1 for country-specific details).

Fig. S1 depicts the geographic coverage of our sample. It shows the number of observations as a share of population (in ~~%~~ percent) by sub-national region for each country in our sample. Our sample achieved broad geographic coverage with observations in 92~~%~~ percent of the sub-national regions and in 61~~%~~ percent of the localities of countries in our sample (see Table S1).

We recruited participants who were of 18 years of age and above~~old and over~~ for the survey using paid advertisement posts on Facebook and Instagram. We used keywords with broad appeal, such as futbol (soccer) or names of local celebrities, to avoid selecting participants based on COVID-19 knowledge or interest. The Supplementary Materials contains additional information about the social media campaigns and shows the posts that were used for Uruguay.

The questionnaire was largely standardized across the countries to allow pooling the data across countries. The primary objective of the ~~surveys~~survey was to measure the negative

economic and well-being impacts of the current pandemic on households in Latin

America and the Caribbean. For this reason, the questionnaire focused on collecting data on labor market outcomes, financial situation~~,~~ and social program enrollment. In addition, the survey collected information on hunger, shortages of key goods~~,~~ and agreement with different policies to slow the spread of COVID-19. A copy of the questionnaire can be found in the Supplementary Material~~-~~section.

Data Validation

To validate ~~our~~the data set, we ~~compare~~compared the demographic characteristics using data from the online and nationally representative household surveys, collected through field visits. Columns (1) and (2) in Table S3 ~~show~~ shows that although the respondents of the online surveys are more educated and more likely to be females, they do not differ substantially in terms of household structure and income levels. Second, Columns (3) and (4) conduct an out-of-sample validation exercise and show that by re-weighting the online responses by the inverse probability of being in the nationally representative sample, the differences in demographic characteristics vanish. The Supplementary Material describes the steps to estimate weights for the online survey and the validation exercise.

In most of ~~our~~the analysis, we re-weight observations in ~~our~~the sample to achieve national representativeness (See Supplementary Material). These estimates also weight observations according to country population to account for differences in sample size across countries. The exception is Fig. 3, which re-weights observations to account for temporal changes in the sample. See Supplementary Material for estimation details. All results are robust to not using weights at all as shown in Figs. S2-S4 and Table S4.

Results

~~We document that the~~ COVID-19 pandemic has negative economic impacts for a broad segment of the population and ~~that~~ these impacts are magnified for those with lower income prior to the pandemic. Overall, 45~~%~~ percent of respondents report that a household member lost a job and~~,~~ among households owning small family businesses, 58~~%~~ percent of respondents reported that a household member closed their business. The recall period for these questions was randomized between 1 week, 2 weeks, and 1 month. We find that the job-loss rates range between 42~~%~~ percent for a recall period of one week to 47~~%~~ percent for a recall period of a month. ~~We~~The result also find that the rates of business closures remain constant at 58~~%~~ percent across recall periods. Compared to similar statistics in the United States (43~~%~~ percent in the case of small businesses [14]), we find greater rates of business closure, implying that the economic impacts of the pandemic may be stronger in developing countries.

These overall effects obscure highly unequal impacts across income levels prior to the pandemic. After accounting for fixed factors by country, the percentage of households reporting job losses declines monotonically with income in January 2020, prior to the onset of the pandemic (Fig. 1). In the case of business closures, the decline is similar though less dramatic. Households reporting income of less than the national monthly minimum wage for January 2020 experienced the largest impacts, with nearly 71~~%~~ percent reporting that a household member lost their job and 61~~%~~ percent reporting that a household member closed their business. This contrasts sharply with the impacts reported by respondents with the highest household incomes. Among the highest income respondents, only 14~~%~~ percent reported that a household member lost their job and 54~~%~~ percent reported that a household member closed their business.

One explanation for these patterns is that high levels of informality in the region

may limit the ability of the most-vulnerable households to keep their sources of income. Using the share of self-employed workers as a proxy for the share of informal workers in the labor market, Figure 2 shows that the labor market informality is positively

Fig 1. Higher rates of job loss and business closure among households in the lowest income group. Point estimates and 95~~%~~ percent confidence intervals for regressing outcome on income bin indicators and country fixed effects. Data is weighted using within- and cross-country weights. See Empirical Methods in Supplementary Material for details.

correlated with loss of livelihood. Since informality rates are high in most developing countries, this result provides a novel explanation of why labor markets in developing countries are particularly hard hit during the crisis.

Fig 2. Higher rates of livelihood loss in countries with higher informality Each dot represents the share of respondents who report that a household member lost a job or closed a business. Data is weighted using within- and cross-country weights.

~~Our~~The data suggest two important explanations for the higher vulnerability of households in developing countries with high levels of informality. First, the type of policies to prevent the spread of ~~the~~ virus affect informal workers more. ~~Our~~The survey captures data from countries without enforced mobility-restriction policies or curfews such as Uruguay, but also more-stringent policies such as mandatory quarantines and closures of non-essential businesses such as the case of Bolivia or Peru. As most of the informal and self-employed workers tend to work in jobs that are prone to contact with other people such as those in the retail or services sectors, as opposed to office or industry jobs, the latter set of policies may lead to larger disruptions in labor markets. Indeed, our sample indicates that the share of respondents reporting job losses in their household during April (69~~%~~ percent) is substantially higher in countries with national or local mandatory quarantines, relative to those of countries that did not implemented mobility-restriction measures (34~~%~~ percent) or only implemented curfews (54~~%~~ percent). See Supplementary Information sections for a list of countries by type of policy.

Second, differences in the ability to telework could also be one reason that the negative impacts of the pandemic are concentrated among households with lower incomes. Among respondents that are still employed, we find that the share of respondents that report working from home during the past week is monotonically increasing with household income in January 2020. Thirty percent of workers from households with incomes below the national minimum wage reported working from home, while 76~~%~~ percent of workers from the highest-income households report working from home.

Impacts on job loss and business closure translate into reductions in income. Table 1 reports within-locality changes in outcomes as a response to job loss or business closure. Column 1 of Panel A shows that respondents who reported a job loss or business closure are 24 percentage points (p-value<0.01) more likely to report a reduction in income. Overall, 71~~%~~ percent of respondents report that they expect their household income in April 2020 to be lower than their household income in January 2020. Thirty-one percent of respondents reported household income less than the national monthly minimum wage for January 2020 and 56~~%~~ percent of households report that they expect their household income to be less than the national minimum wage in April 2020. Fig. 3 shows that the distribution of household income expected in April 2020 is a leftward shift of the distribution of household income in January 2020. In particular, the share of households with incomes marginally above the national minimum wage declines between January and April 2020, suggesting that many vulnerable households expect to fall into poverty.

~~We~~The study find that job loss and business closure lead to reductions in health and food security. Columns 2-3 of Panel A of Table 1 ~~show~~shows that households with a job loss or

business closure are 12 percentage points (p-value <0.01) more likely to suffer from

Table 1. The loss of livelihood during the pandemic is linked to changes in nutrition and policy support

Panel A: Impacts on income, food security, and health

	(1) Decreased income	(2) Went hungry	(3) Eats less healthy
Lost job or closed business	0.241***	0.127***	0.085***
	(0.008)	(0.008)	(0.008)
Observations	186,058	198,190	173,956
Adjusted R2	0.487	0.602	0.430

Panel B: Impacts on transfers and policy support

	(1) Gift/Loan	(2) Gov. Priority	(3) Lockdown (>=month)
Lost job or closed business	0.225***	-0.027***	-0.042***
	(0.008)	(0.008)	(0.010)
Observations	198,017	196,076	125,359
Adjusted R2	0.479	0.482	0.540

$*p < 0.1, **p < 0.05, ***p < 0.01$

The table reports regression coefficients capturing the relationship between livelihood losses during the pandemic and outcomes. Each column reports results of a regression of the dependent variable on an indicator of whether any household member either lost a job or closed a business and a vector of covariates. In addition, all regressions control for locality $\times$ day of survey completion fixed effects (18,764), as well as economic-sector fixed effects. Standard errors are clustered at the locality level (3,165). Data is weighted using within- and cross-country weights. See Empirical Methods in Supplementary Information Appendix for details.

Fig 3. The share of households in the bottom part of the income distribution is expected to increase. Shares of households in each income bin for incomes reported for January 2020 and April 2020. Data counts weighted using within- and cross-country weights. See Empirical Methods in Supplementary Material for details.

hunger and 8 percentage points (p-value<0.01) more likely to have a less healthy diet relative to their diet prior to the pandemic. The results suggest that the impacts of the pandemic may have long-lasting consequences linked to declines in the stock of human capital. Consistent with the results from Figure 2, we also find that the consequences of the loss of livelihoods on food security are stronger in countries with higher levels of informality (See Table S5). This suggests that the structure of labor markets not only magnified the exposure to job losses and business closures, but also magnified the impacts of the loss of livelihoods on household welfare as informal workers may have lower access to formal safety nets.

~~We~~The study find that households cooperate across income levels to smooth the negative economic impacts of the pandemic. Seventy percent of respondents who reported January 2020 household income less than the national minimum wage also reported that a household member received a gift or loan from a friend or relative. In contrast, only 26~~%~~ percent of respondents with the highest incomes in January 2020 report that a household member received a gift or loan from a friend or relative. This pattern reverses for providing a gift or loan. Thirty percent of households with January 2020 income less than the national minimum wage reported that a household member provided a gift or loan compared to 57~~%~~ percent of households with the highest incomes January 2020. Across all income categories, the reception of a gift or transfer is concentrated among households that reported losing a job or closing a business. Column 1 of Panel B of Table 1 shows

that households that lost their livelihoods during the crises are 22 percentage points (p-value<0.01) more likely to receive transfers from relatives or friends. This highlights the importance of informal social protection networks as a tool to cope with the negative impacts of the pandemic.

Despite these substantial economic impacts, there is strong support for measures to slow the spread of the coronavirus. Overall, 77~~%~~ percent of respondents agree with a statement that the top priority of the national government should be to stop the pandemic and 54~~%~~ percent of respondents think that non-essential businesses should be closed for one more month. Although the support for these policies is broad, the support is likely to decrease as more households lose their livelihoods. Column 2 of Panel B from Table 1 shows that the probability of agreeing with the idea that the Government's priority should be fighting the pandemic is 2 percentage points (p-value<0.01) lower among households that experienced a job loss or business closure. Column 3 of Panel B shows that the loss of livelihoods during the pandemic is linked to a 4 percentage-point decline (p-value<0.01) in the probability of agreeing with closing non-essential business for one more month. Thus, the support for policies to slow the spread of the coronavirus is fragile. Indeed, Fig. 4 shows that~~,~~ as days go by, the decline in support for keeping businesses closed for one more month declines faster among households that lose their livelihoods. One important implication is that, without further assistance to impacted households, compliance with mobility restriction policies is likely to decline.

Fig 4. Support for extending lockdown policies declines more among households that lost their livelihoods. Point estimates and 95~~%~~ percent confidence intervals for the share of respondents supporting extending lockdown policies according to days since first COVID-19 case in the country. Data is weighted using within~~-~~ and cross-country weights. See Empirical Methods in Supplementary Material for details.

Data Analysis

Figure 1

In Fig. 1 ~~we show~~ shows how job loss and business closures vary throughout the income distribution. Job loss is a dummy variable that equals one if the respondent indicates that they lost their job within the last week, two weeks or month (these horizons were randomly assigned). Similarly, the variable for business closures equals one if subjects report that their business was forced to close by the government or by lack of demand within the same randomly assigned time horizon.

To explore differences in job loss and business closure across the income distribution, we first aggregate responses at the country and income bracket level using within-country weights as described in the Supplementary Information Appendix. We then regress by OLS~~,~~ the country averaged outcome on indicators for income bin and control for country fixed effects, weighting by country population. Because we only have 17 countries in our sample, using standard clustered standard errors would result in incorrect inference. Therefore, we follow [20] by aggregating the data at the country level by income category. Fig. 1 shows the point estimates and 95~~%~~ percent confidence intervals for each income bin average. As robustness, we show results for the raw pooled data, without applying weights in Figure S2.

Figure 2

We present a cross-country comparison of the shares of households who lost their livelihoods as a function of the share of self-employed workers in the economy. For this,

we identified the respondents ~~that~~who reported that at least one household member lost their job ~~during~~ or had to close their businesses during the survey reference period, and we obtained shares using weights to correct for sampling issues (See Supplementary Information section). Data corresponding to the share of self-employed workers was obtained from the World Bank's World Development Indicators.

Figure 3

We present the share of household income in each income bin for incomes reported by respondents corresponding to January 2020 and April 2020 in Fig. 3. We restrict the sample to responses collected between April 13th to May 1st 2020 to include all countries. Shares are calculated by re-weighting counts by within~~-~~ and across-country weights as detailed in Supplementary Material section Estimation of weights. As robustness, we show the results for the raw pooled data~~,~~ without applying weights in Figure S3.

Figure 4

Fig. 4 shows the share of respondents supporting extending the lockdown policies by at least one month over time since the first COVID-19 case in the country. One challenge to explor~~ing~~e how support changes over time is that the timing of responses varies between countries~~,~~. ~~therefore~~Therefore, over time we have new countries entering the sample, and the change in sample composition could bias our results. We address this issue in two steps. First, we construct new weights and re-weight respondents after day 31 to match those responding at the beginning of the series (days 30 and 31) on observable demographic characteristics.

We estimated a model by fitting a logistic function (logit) and computed predicted probabilities of having responded to the survey on day 30 or 31 relative to first case. As explanatory variables we include demographic characteristics such as household size, age indicators for: presence of children or elderly, having felt hunger, woman, education: primary or less, college education or higher, household income categories (<1, 1-2,2-3, 8-11,>11), and for day of week. We then used inverse probability weights based on the propensity score obtained.

Second, we separately estimate OLS regressions for respondents whose household member lost job or closed business and for those who did not, where the dependent variable is an indicator variable that equals one if they support extending the lockdown by at least a month, and zero otherwise. The independent variables are indicators for days since first COVID-19 case, day of week indicators, and country fixed effects. The regression is weighted by the inverse probability weight described in step one and we employ robust standard errors. Fig. 4 presents the point estimates and 95~~%~~ percent confidence intervals for these estimates. We present similar estimates when using the pooled raw data without re-weighting in Figure S4.

Table 1

In Table 1, we report comparisons of differences in the relevant outcomes between respondents whose households experienced a loss of livelihood during the pandemic period. To control for time-varying characteristics we perform comparisons focusing on respondents in the same locality and who completed the survey during the same day. We exploit granular data from over 3000 localities in 17 countries. Thus, we focus our analysis in the subset of 18,000 locality-date-of-response cells that include more than one observation. This approach allows us to isolate time-varying locality shocks, and thus purge regional confounding factors. In addition we control for industry sector fixed

effects, to prevent differences in the sectors related to the household's main economic activities from driving the results, as exposure to the effects of the pandemic may vary across sectors.

We operationalize our approach by estimating the following specification, which is similar to that used in reference [21]:

$$\mathrm{Y}_{\mathrm{i,l,c,t}} = \beta \mathrm{Lostlivelihood}_{\mathrm{i,l,c,t}} + \mathbf{X}_{i,c,l,t}\Sigma + \delta_{l,c,t} + \theta_s + s_{i,l,c,t} \tag{1}$$

Here, $Y_{i,l,c,t}$ denotes the outcome of interest corresponding to respondent i in locality l from country c collected in date t. Lost livelihood$_{i,l,c,t}$ is an indicator of whether any member in the respondent's loss her/his job or closed her business during the the past week, two-weeks, or month. $\mathbf{X}_{i,c,l,t}$ is vector of demographic characteristics of the respondent (age, education level and gender) and of household characteristics (household size, presence of children younger than 5, presence of school-age children, and people 60 years old or older). $\delta_{l,c,t}$ denotes locality-date fixed effects, and θ_s denotes industry fixed effects based on the main pre-pandemic source of income of the respondent's household. $s_{i,c,l,t}$ denotes unobserved shocks. To account for possible serial correlation of outcomes within localities, we cluster the standard errors at the locality-country level.

The parameter of interest, β, is reported in Table 1 and captures within-locality differences in outcomes between respondents whose households experienced a loss of livelihood during the pandemic and those who didn't. The reported models are estimated using weights to achieve country-level representativeness, to correct for differences in sample size across countries, and to provide higher based on country population size. All results are robust to excluding these weights (See Robustness Section in Supplementary Material).

To investigate whether the correlates of job loss or business closures and household outcomes are stronger in countries with high levels of informality, we also estimate the following specification:

$$\begin{aligned}\mathrm{Y}_{\mathrm{i,l,c,t}} = {} & \beta_1 \mathrm{Lostlivelihood}_{\mathrm{i,l,c,t}} + \beta_2 \mathrm{Lostlivelihood}_{\mathrm{i,l,c,t}} \times \mathrm{Self-employment}_c \\ & + \mathbf{X}_{i,c,l,t}\Sigma + \delta_{l,c,t} + \theta_s + s_{i,l,c,t}\end{aligned} \tag{2}$$

Where *Self-employment* denotes the share of self-employed workers in country c and was obtained from the World Bank's World Development Indicators, using the most-recent observations for each country. We also report results using the share of informal workers (as a share of non-agricultural workers) in each country. This information was not available for Suriname, Jamaica, Trinidad and Tobago, Barbados, and Bahamas. See results in Table S4.

Dependent variables: *Went hungry* is an indicator of whether any household member went hungry during the past week due to lack of food. *Eats less healthy* takes the value of one if the respondent somewhat or totally agrees with the statement "I eat more unhealthy foods than normal". *Gifts/Loans* is an indicator of whether the respondent's household received a gift or transfer from either friends or relatives during the preceding week. Gov. Priority is an indicator of whether the respondent somewhat agrees or totally agrees with the statement "The government's priority should be to stop the spread of the pandemic". *Lockdown (>= month)* is an indicator of whether the respondent reports agreeing with closing non-essential business for one month or longer. As this question was asked only to people that reported agreeing with policies that require non-essential businesses to close, *Lockdown(>= month)* takes the value of zero when the respondent reported not supporting measures of keeping non-essential businesses closed at all, regardless of the time.

Robustness

We demonstrate that all our results are robust to not re-weighting observations. Reassuringly all results are both qualitatively and quantitatively similar. See SI Appendix, Figs. S2-S4 and Panel A of Table S4 for unweighted results. In addition, Panels B and C of Table S4 report robustness related to using only country-date fixed effects instead of locality-date fixed effects as controls, with and without weights. Reassuringly our results are both quantitatively and qualitatively similar.

Conclusions

These results show that the negative economic impacts of the COVID-19 pandemic have been concentrated among those with lower incomes prior to the pandemic. This finding is important from both social and economic perspectives. Inequality is an important social outcome itself and also has important economic implications. Although further research is needed, several studies have found that current inequality is negatively correlated with future economic growth [22, 23], and in particular, inequality driven by the lower tail of the income distribution stunts economic growth [24]. This implies that the unequal economic impacts of this short-term public health pandemic could have long-term implications for economic growth. In addition, the result of the study indicates ~~our results indicate~~ that country-level rates of informality in labor markets are linked to stronger negative impacts and low resilience, suggesting that prompt policy reactions targeted at protecting informal workers is needed.

Supporting information

S1 Fig. Sample has broad geographic coverage at the sub-national level. The sub-national regions of each country in the sample are shaded according to number of observations as a share of population (in ~~%~~percent). Sources of population data for each country are shown in the Supplementary Text Information Section.

S2 Fig. Higher rates of job loss and business closure among households in the lowest income group. Point estimates and 95~~%~~ percent confidence intervals for regressing outcome on income bin indicators and country fixed effects. Pooled data (no weights), robust standard errors. See the Empirical Methods section in the main text for more details.

S3 Fig. The share of households in the bottom part of the income distribution is expected to increase. Shares of households in each income bin for incomes reported for January 2020 and April 2020. Pooled data (no weights). See the Empirical Methods section in the main text for more details.

S4 Fig. Support for extending lockdown policies declines more among households thatlost their livelihoods. Point estimates and 95~~%~~ percent confidence intervals for the share of respondents supporting extending lockdown policies according to days since first COVID-19 case in the country. Pooled data (no weights). See the Empirical Methods section in the main text for more details.

S1 Table. Date of Launch and Number of Observations by Country.

S2 Table. Parameters of logit models for the probability of being in the nationally representative survey.

S3 Table. Differences between Online Survey Data and Household (field) Survey Data.

S4 Table. Loss of livelihoods and changes in well-being, and policy support (unweighted results).

S5 Table. Loss of livelihoods and changes in well-being, by labor-market characteristics.

Acknowledgments

We would like to thank Sebastian Espinoza and Maria Paula Medina for superb research assistance. We would like to thank Julián Cristia for his encouragement and advise with the project. We also want to thank Tom Sarrazin, Sebastián Oliva and Pablo Bachelet for their tremendous support in the dissemination of the survey. We also want to thank the support of IDB's country offices, country representatives and country economists for their invaluable input in customizing the questionnaires for each country. In particular, we want to thank Marta Ruiz Aranz and Diether Beuermann for their support in the rollout of the surveys in Central America and the Caribbean. We would like to thank Ana María Ibañez, Eric Parrado, Michael Lovenheim, and the team of researchers in IDB's Research Department for valuable support and feedback in different parts of the project. Opinions, findings, conclusions and recommendations expressed here are those of the author and do not necessarily reflect the views of the Inter-American Development Bank.

References

1. Stiglitz JE. Why It Matters. In: The priceof inequality: How today's divided society endangers our future. W.W. Norton and Company; 2013.

2. Milanovic B. Global Inequality in This Century and the Next. In: Global inequality: A new approach for the age of globalization. Harvard University Press; 2016.

3. Piketty T, Yang L, Zucman G. Capital accumulation, private property, and rising inequality in China, 1978-2015. American Economic Review. 2019;109(7):2469–2496.

4. Zucman G. Global wealth inequality; 2020.

5. Smeeding T. Income, wealth, and debt and the Great Recession; 2012.

6. Heathcote J, Perri F, Violante GI. Unequal we stand: An empirical analysis of economic inequality in the United States, 1967-2006. Review of Economics Dynamics. 2010;13(1):15–51.

7. Brzezinski M. Income inequality and the Great Recession in Central and Eastern Europe. Economic Systems. 2018;42(2):219–247.

8. Blackman A, Ibáñez AM, Izquierdo A, Keefer P, Moreira MM, Schady N, et al. La polı́tica publica frente al Covid-10; 2020. IDB Policy Note.

9. Fetzer T, Witte M, Hensel L, Jachimowicz JM, Haushofer J, Ivchenko A, et al. Global behaviors and perceptions at the onset of the COVID-19 pandemic; 2020.

10. Allcott H, Boxell L, Conway JC, Gentzkow M, Thaler M, Yang DY. Polarization and public health: Partisan differences in social distancing during the coronavirus pandemic; 2020. NBER Working Paper No. 26946.

11. Briscese G, Lacetera N, Macis M, Tonin M. Compliance with COVID-19 social-distancing measure in Italy: The role of expectations and duration; 2020. NBER Working Paper No. 26916.

12. Bento AI, Nguyen T, Wing C, Lozano-Rojas F, Ahn YY, Simon K. Evidence from internet search data shows information-seeking responses to news of local COVID-19 cases. Proceedings of the National Academy of Sciences. 2020;Available from: https://www.pnas.org/content/early/2020/05/01/2005335117.

13. Adams-Prassl A, Boneva T, Golin M, Rauh C. Inequality in the impact of the coronavirus shock: Evidence from real time surveys; 2020.

14. Bartik AW, Bertrand M, Cullen ZB, Glaeser EL, Luca M, Stanton CT. How Are Small Businesses Adjusting to COVID-19? Early Evidence from a Survey. National Bureau of Economic Research; 2020. 26989.

15. Alon TM, Doepke M, Olmstead-Rumsey J, Tertilt M. The Impact of COVID-19 on Gender Equality. National Bureau of Economic Research; 2020. 26947.

16. Messina J, Silva J. Wage inequaity in Latin America. Understanding the past to prepare the future; 2018.

17. Levy S, Schady N. Latin America's social policy challenge: Education, social insurance, and redistribution. Journal of Economic Perspectives. 2013;27(2):193–218.

18. Amarante V, Galván M, Mancero X. Inequality in Latin America: A global measurement; 2016.

19. Báez JE, Fuchs A, Rodríguez-Castelán C. Shaking up economic progress. Aggregate shocks in Latin America and the Caribbean; 2017.

20. Bertrand M, Duflo E, Mullainathan S. How Much Should We Trust Differences-In-Differences Estimates?*. The Quarterly Journal of Economics. 2004 02;119(1):249–275.

21. Gertler P, Gruber J. Insuring Consumption Against Illness. American Economic Review. 2002 March;92(1):51–70.

22. Berg AG, Ostry JD. Inequality and Unsustainable growth: Two sides of the same coin?; 2011.

23. Ostry J, Berg A, Tsangarides CG. Redistribution, inequality, and growth; 2014.

24. Cingano F. Trends in income inequality and its impact on economic growth; 2014.

Click here to download Figure Figure1.eps

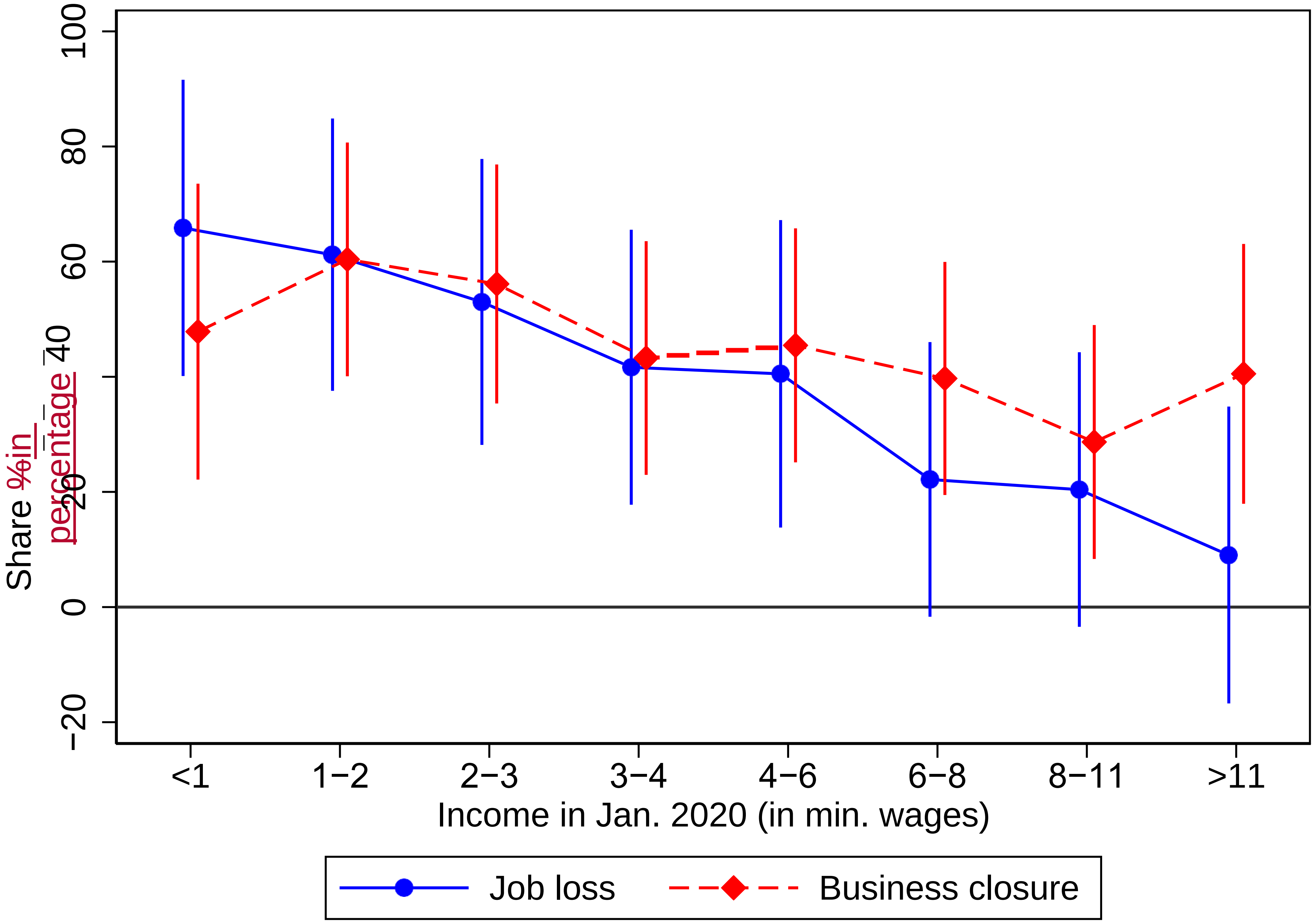

Click here to download Figure Figure2.eps

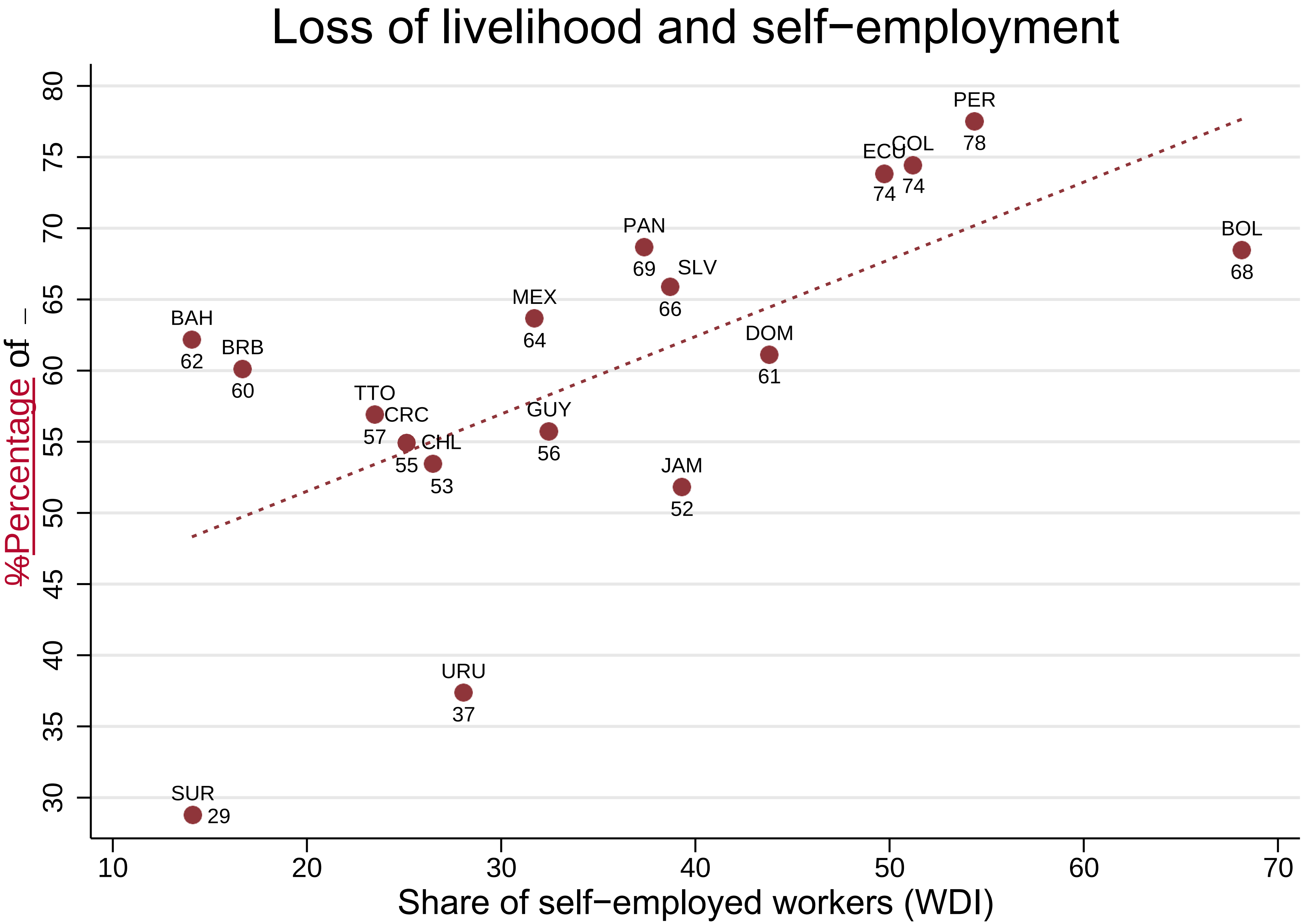

Click here to download Figure Figure3.eps

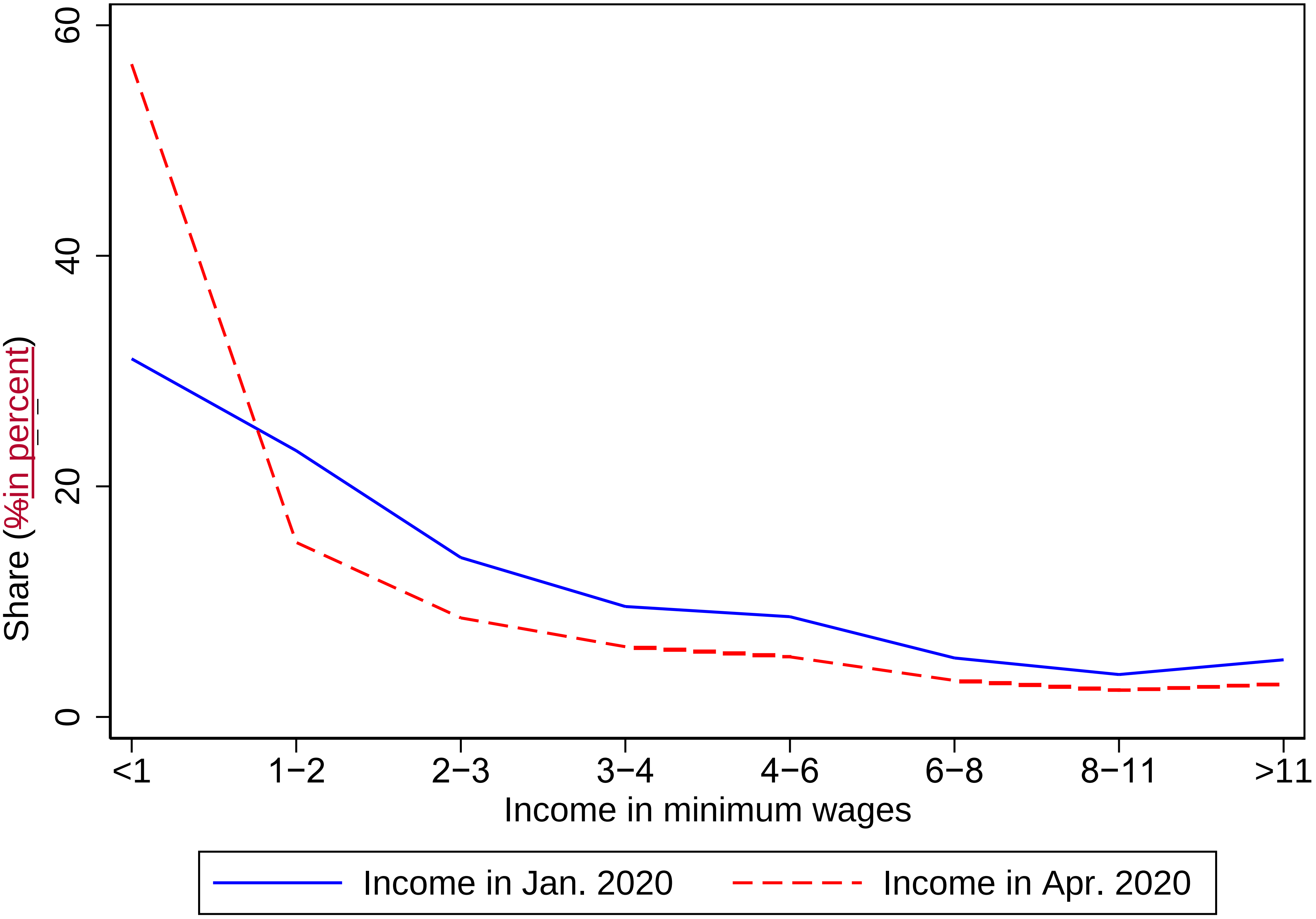

Click here to download Figure Figure4.eps

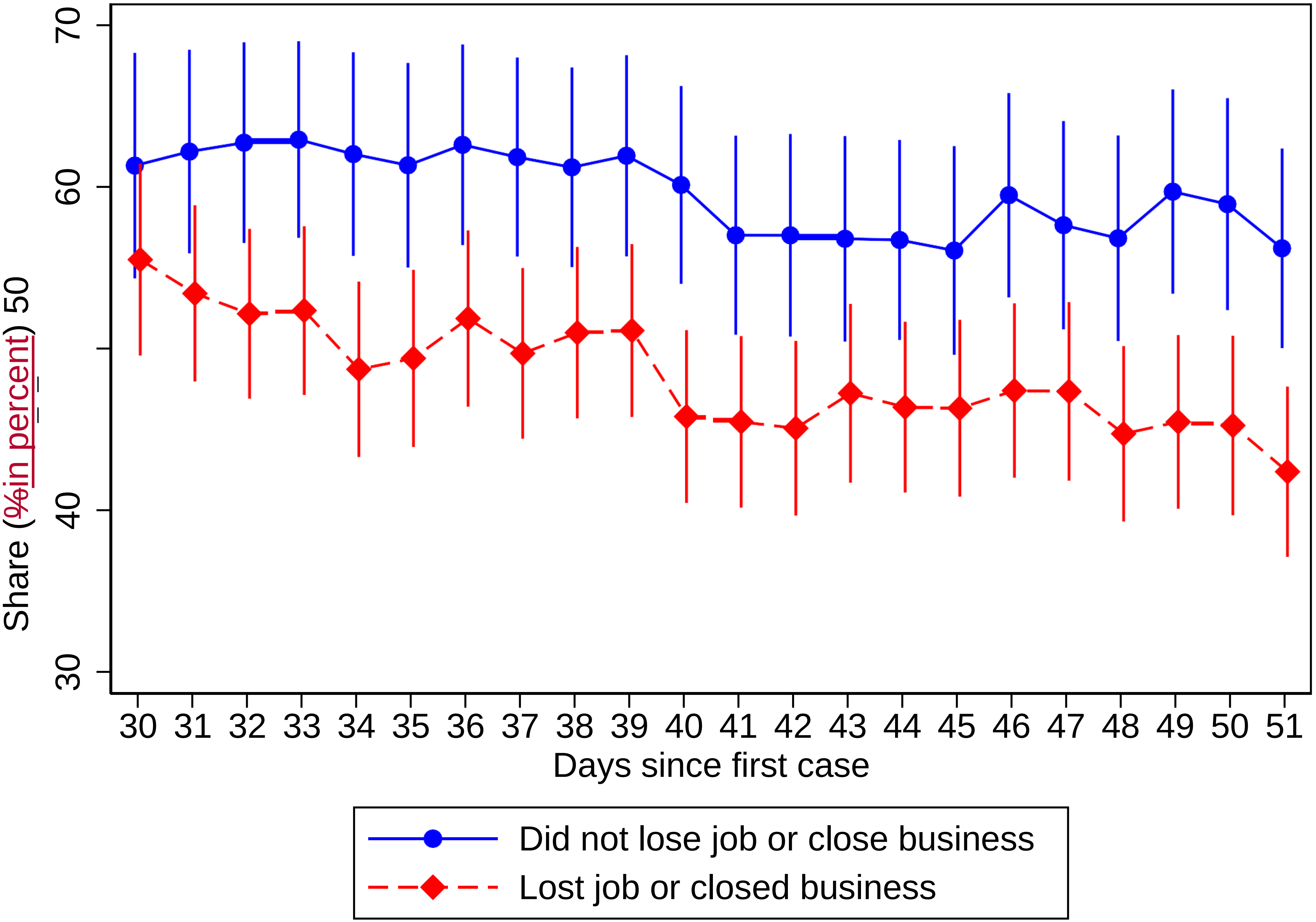

Click here to download Figure SFigure1.eps

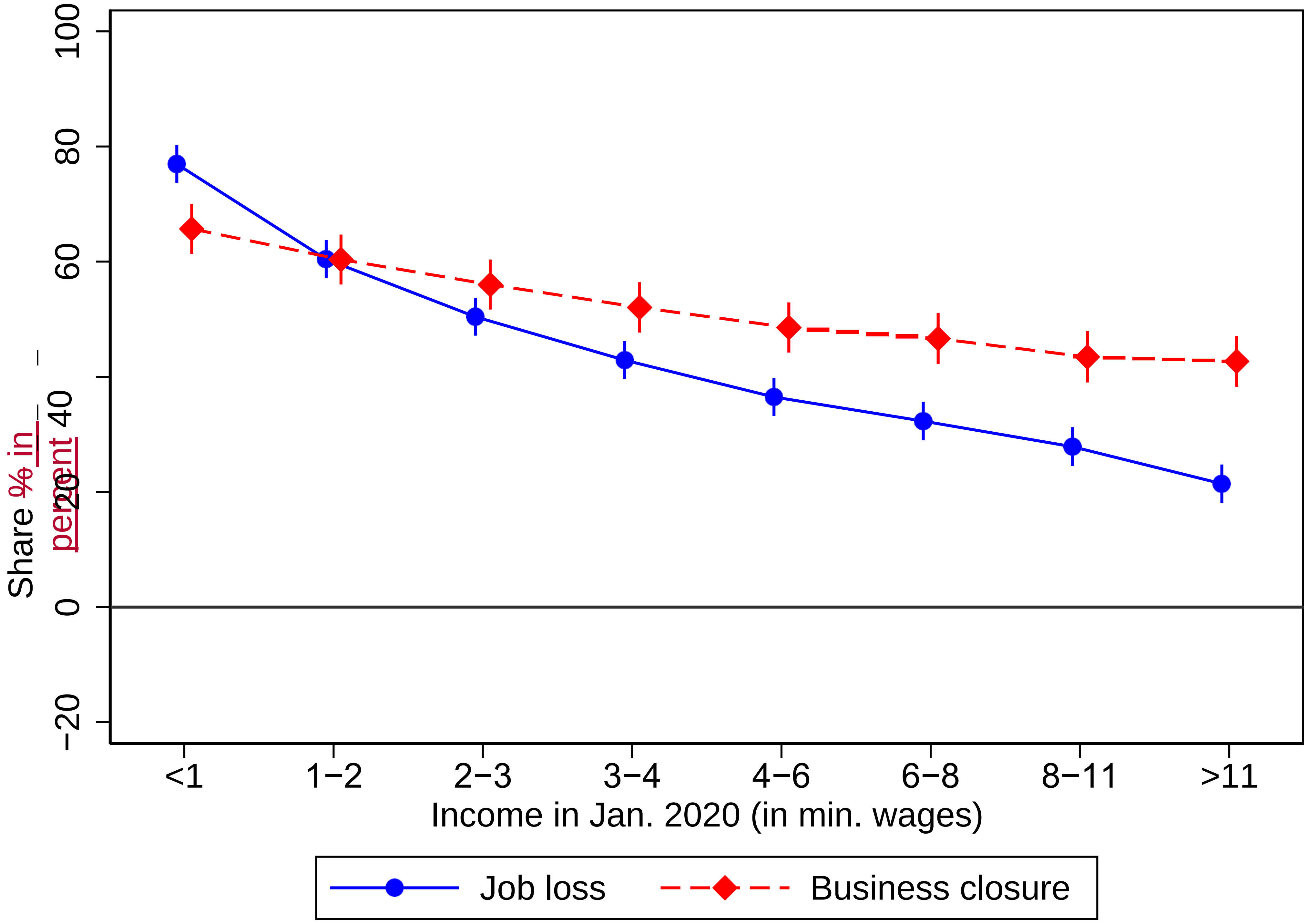

Click here to download Figure SFigure2.eps

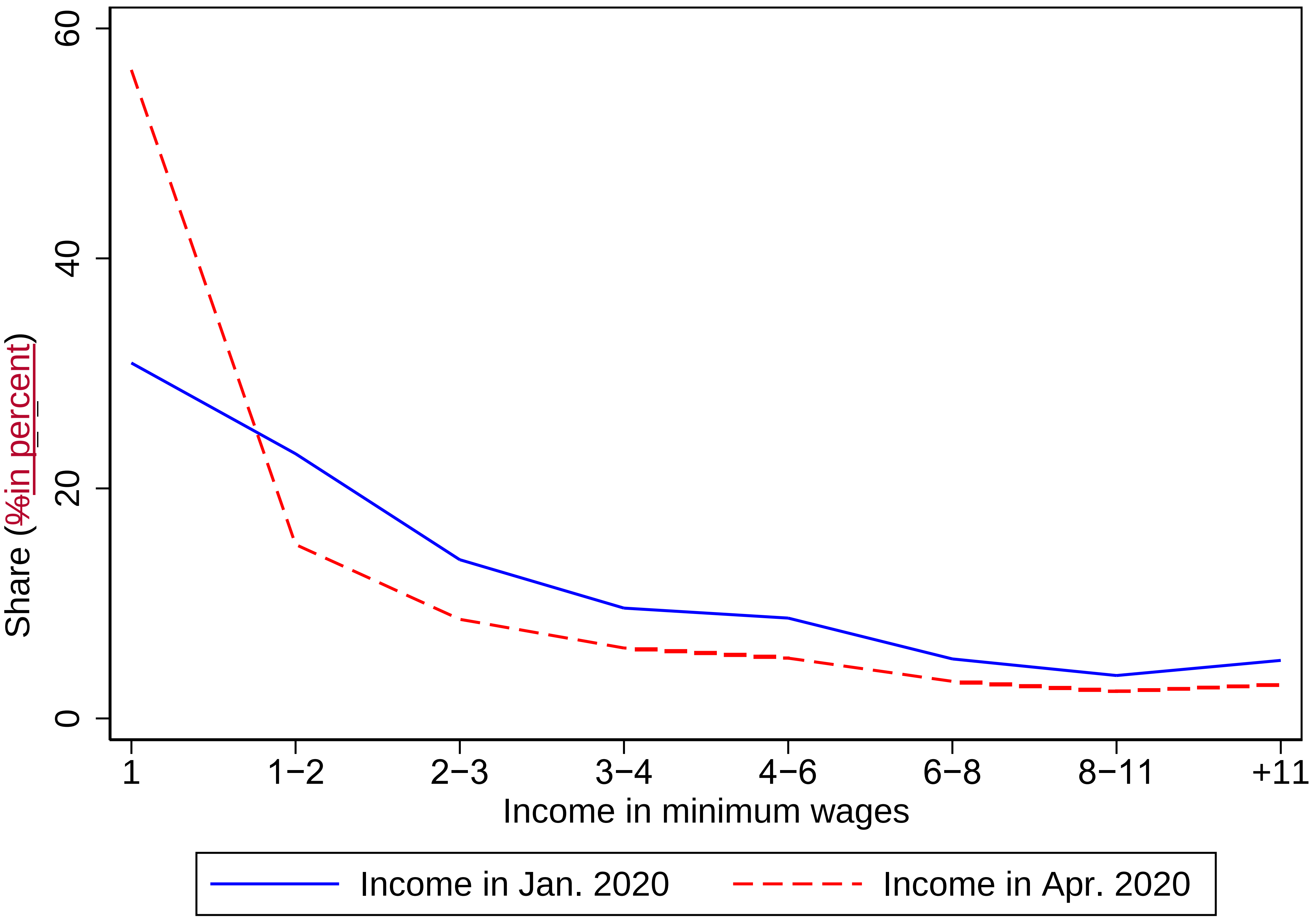

Click here to download Figure SFigure3.eps

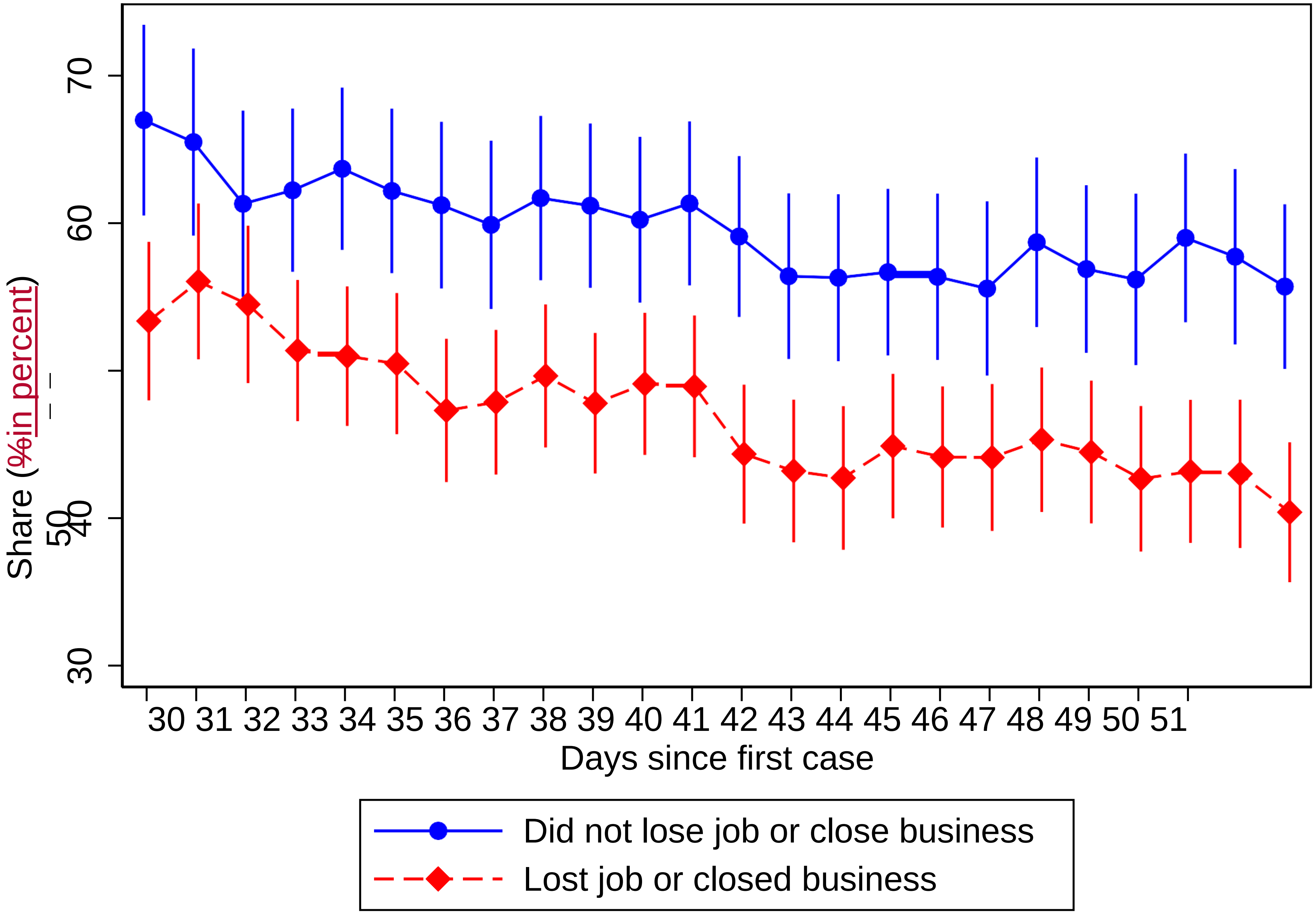

Click here to access/download
Supporting Information
PLOS_ONE_Covid19-Supporting Information.pdf
