# Supplementary material for: The unequal impact of the coronavirus pandemic: Evidence from seventeen developing countries
Source: PLoS One. 2020 Oct 7;15(10):e0239797. doi: 10.1371/journal.pone.0239797 (PMC7540865; doi:10.1371/journal.pone.0239797)
Supplement: Attachment — Submitted filename: Response to Reviewers.pdf [file pone.0239797.s003.pdf]

Responses to the Editor

PONE-D-20-15019, "The unequal impact of the coronavirus pandemic: Evidence from seventeen developing countries"

PLOS ONE

We would like to thank the editor for the constructive comments and questions. Please find our detailed responses to each of the questions raised below:

Q1. *Please ensure that your manuscript meets PLOS ONE's style requirements, including those for file naming.*

> **R1.** We have reviewed the style requirements and believe to have implemented changes to meet PLOS ONE style requirements. If we missed anything please let us know and we would be happy to rectify it.

Q2. *Thank you for stating the following in the Financial Disclosure section:*

"Bridget Hoffmann and Diego Vera-Cossio were joint recipients of the Inter-American Development Bank's Coronavirus research funds (RG-E1700) - www.iadb.org. The funders had no role in study design, data collection and analysis, decision to publish, or preparation of the manuscript."

We note that one or more of the authors are employed by a commercial company: Inter-American Development Bank.

> **R2.** The Inter-American Development Bank is not a commercial company. It is an international organization (like the World Bank or Asian Development Bank). It is the largest source of development financing for Latin America.
>
> Nevertheless, we have followed suggestions raised in 2.1 and 2.2 by amending the information provided in the Funding Statement clarifying that the organization did not play a role in any aspects of the study (design, data collection, analysis, decision to publish or preparation of the manuscript):
>
> > "This project was funded by the Inter-American Development Bank's Coronavirus research funds (RG-E1700 - Coronavirus survey). The funder provided support in the form of salaries for authors Bridget Hoffmann and Diego Vera-Cossio, and provided funds to cover the cost of recruiting subjects via social media advertising, but did not have any additional role in the study design, data collection and analysis, decision to publish, or preparation of the manuscript. The specific roles of these authors are articulated in the 'author contributions' section."

Furthermore, we included a Competing Interests Statement declaring affiliation of the authors to this organization and clarifying that the affiliation does not alter adherence to all PLOS ONE policies on data sharing and materials:

> "The authors Bridget Hoffmann and Diego A. Vera-Cossio are employees in the Research Department at the Inter-American Development Bank. The author Nicolas Bottan is employed at Cornell University.
>
> The authors have no relevant financial or non-financial competing interests to declare. Our affiliations do not alter our adherence to PLOS ONE policies on sharing data and materials."

Q3. We note that you have stated that you will provide repository information for your data at acceptance. Should your manuscript be accepted for publication, we will hold it until you provide the relevant accession numbers or DOIs necessary to access your data. If you wish to make changes to your Data Availability statement, please describe these changes in your cover letter and we will update your Data Availability statement to reflect the information you provide.

R3. We have amended our Data Availability statement and cover letter to include access information to data and replication materials. All material has been uploaded to the Harvard Dataverse and is available at: https://doi.org/10.7910/DVN/7WX5UU

Q4. *We note that Supporting Information includes images of participants in the study.*

As per the PLOS ONE policy (http://journals.plos.org/plosone/s/submission-guidelines#loc-human-subjects-research) on papers that include identifying, or potentially identifying, information, the individual(s) or parent(s)/guardian(s) must be informed of the terms of the PLOS open-access (CC-BY) license and provide specific permission for publication of these details under the terms of this license. Please download the Consent Form for Publication in a PLOS Journal (http://journals.plos.org/plosone/s/file?id=8ce6/plos-consent-form-english.pdf). The signed consent form should not be submitted with the manuscript, but should be securely filed in the individual's case notes. Please amend the methods section and ethics statement of the manuscript to explicitly state that the patient/participant has provided consent for publication: "The individual in this manuscript has given written informed consent (as outlined in PLOS consent form) to publish these case details".

If you are unable to obtain consent from the subject of the photograph, you will need to remove the figure and any other textual identifying information or case descriptions for this individual.

R4. We believe this is referring to images presented in the Supplementary Information section on the description of Recruitment Materials. Note that these are not images of subjects, but rather stock images used in recruitment ads. However, we removed these images to comply with this point and Q5 below.

Q5. *You may seek permission from the original copyright holder of Figure S1 to publish the content specifically under the CC BY 4.0 license.*

R5. We created Figure S1 using our survey and administrative data, and shapefiles obtained from GADM. GADM distributes the shapefiles openly for academic use (https://gadm.org/data.html). We have included the following in the notes to Figure S1:

"Administrative boundary shapefiles were obtained from the GADM database (www.gadm.org)."

This is similar to the way these boundary files have been used and cited in other PLOS ONE publications. See for example:
https://journals.plos.org/plosntds/article/figures?id=10.1371/journal.pntd.0006585
or https://journals.plos.org/plosone/article/figures?id=10.1371/journal.pone.0209641

Responses to Referee #1

PONE-D-20-15019, "The unequal impact of the coronavirus pandemic: Evidence from seventeen developing countries"

PLOS ONE

We thank the referee for their careful and constructive feedback. We have completed the revision implementing the suggested changes carefully. By addressing the issues raised, we believe the article has significantly improved overall. We hope the referee agrees with this. Please find our detailed responses to each of the questions raised below:

Q1. *The original research article has a lot of merit and the information presented is of great value. However, presently the format of the manuscript is not in order. A standard research article is composed of 4 main important components; Introduction (background), Methods, Results and a Discussion. The article has a brief introduction which can be further substantiated by mentioning both the global and regional perspectives by referencing recent relevant published literature.*

R1. Thank you for pointing this out. We have re-arranged the article to reflect these four main sections. Furthermore, the introduction has been expanded and includes the suggested references. Below we reproduce the new paragraph we added to the introduction:

> "It is important to measure the economic impacts of the COVID-19 pandemic on households in Latin America and the Caribbean. [21], for example, describes the potential disruptive effects of the pandemic across a wide domain of the global economy, including on labor supply and the risks of small business closure and unemployment. This study complements this work by quantifying the short-term implications of the pandemic on job losses and business closures and shows that these impacts further aggravate inequality in the region. Furthermore, [22] describes how macroeconomic spillovers can amplify the adverse economic effects of the pandemic, which would suggest that the estimates obtained are a lower bound. The results presented in this study may be useful to inform pandemic mitigation policy; by indicating where the economic impacts of large-scale lockdowns are particularly large [23]."

Corresponding references:

21. Zaman, K Taeen and Islam, Hiya and Khan, Asir Newaz and Shweta, DipitaSaha and Rahman, Ahsab and Masud, Jaasia and Araf, Yusha and Sarkar, Bishajit and Ullah, Md Asad. COVID-19 Pandemic Burden on Global Economy: A Paradigm Shift; 2020.
22. Kohlscheen, Emanuel and Mojon, Benoit and Rees, Daniel. The macroeconomic spillover effects of the pandemic on the global economy. SSRN 3569554. 2020.
23. Gershon, David and Lipton, Alexander and Levine, Hagai. Managing covid-9 pandemic without destructing the economy. arXiv preprint arXiv:2004.10324;2020

Q2. *The methods section needs a description of sampling technique.*

R2. We rearranged and expanded the methods section to include a dedicated subsection for "Sampling and Validation", in which we provide a description of the sampling technique used. Below we reproduce that section for your convenience:

> "The same recruitment methods were followed in all countries. The study recruited participants who were 18 years of age and above using paid advertisements on social media. Participation in the survey was purely voluntary. The advertisements used keywords with broad appeal, such as *fútbol* (soccer) or the names of local celebrities, to avoid selecting participants based on COVID-19 knowledge or interest (details are available in the Supplementary Information section). For each country, we exclude incomplete surveys, surveys in which the respondent's IP address did not belong to that country, surveys flagged as repeated, surveys with invalid responses, and surveys unreasonable completion time from the sample.
>
> The final sample consists of a total of 230,540 completed responses. Fig. S1 depicts the geographic coverage of the sample. It shows the number of observations as a share of population (in percent) by sub-national region for each country. The sample achieved broad geographic coverage, with observations in 92 percent of the sub-national regions (see Table S1).
>
> To validate the representativeness of the data, demographic characteristics from the online survey were compared to nationally representative household surveys. Columns (1) and (2) in Table S3 shows that although the respondents of the online survey are more educated and more likely to be females, they do not differ substantially in terms of household structure or income levels. Columns (3) and (4) conduct an out-of-sample validation exercise and show that by re-weighting the online survey responses by the inverse probability of being in the nationally representative sample, the differences in demographic characteristics vanish. The Supplementary Information section provides details on the steps taken to estimate weights for the online survey and the validation exercise.
>
> In order to document the economic and well-being impacts of the current pandemic is having on households in Latin America and the Caribbean, most of the analysis presented in this study re-weights observations to achieve national representativeness. These estimates also weight observations according to country population to account for differences in sample size across countries. The exception is Fig. 3, which re-weights observations to account for temporal changes in the sample. See the Supplementary Information section for estimation details. All results are robust to not using weights as shown in Figs. S2, S3 and S4 and Table S4."

Q3. *The discussion component is missing and mixed with a extension of results section. the following resource will help in improving quality of reporting the study.*

Q3. Thank you for the reference. We separated the discussion from the results and expanded the discussion. The sections are too long to reproduce here. Additionally, to address your response in question 4 on whether "the manuscript presented in an intelligible fashion and written in standard English?" we did two things. First, we implemented the changes you suggested in Q1 to Q3. Second, we had the text reviewed by a professional copyeditor to ensure that the article is clear, correct, and unambiguous.

Responses to Referee #2

PONE-D-20-15019, "The unequal impact of the coronavirus pandemic: Evidence from seventeen developing countries"

PLOS ONE

We thank the referee for their careful and constructive feedback. We have completed the revision implementing the suggested changes carefully. By addressing the issues raised, we believe the article has significantly improved overall. We hope the referee agrees with this. Please find our detailed responses to each of the questions raised below:

Q1. *Subject to the data availability, sample size selection procedure should be elaborated and clearly written.*

> **R1.** We have expanded the description of the sample selection in the "Sampling and Validation" subsection within the "Methods" section. We reproduce the relevant additions below for your convenience:
>
> > "The same recruitment methods were followed in all countries. The study recruited participants who were 18 years of age and above using paid advertisements on social media. Participation in the survey was purely voluntary. The advertisements used keywords with broad appeal, such as fútbol (soccer) or the names of local celebrities, to avoid selecting participants based on COVID-19 knowledge or interest (details are available in the Supplementary Information section). For each country, we exclude incomplete surveys, surveys in which the respondent's IP address did not belong to that country, surveys flagged as repeated, surveys with invalid responses, and surveys unreasonable completion time from the sample.
> >
> > The final sample consists of a total of 230,540 completed responses. Fig. S1 depicts the geographic coverage of the sample. It shows the number of observations as a share of population (in percent) by sub-national region for each country. The sample achieved broad geographic coverage, with observations in 92 percent of the sub-national regions (see Table S1)."

Q2. *The method section and sampling procedure should be clearly written to fulfill the aim and objective of the study.*

> **R2.** We restructured and rewrote the "Methods" section entirely. In the revised version we separated the Methods section into three subsections:

- Design, Setting and Participants: where we clearly state how the survey employed addresses the intended aim and objective of the study.
- Sampling and Validation: as explained in Q1, we elaborate on sample selection procedures and discuss how we validate the data collected.

- Statistical Analysis: we describe how the collected data is analyzed to fulfill the aim and objective of the study.

We are not reproducing the text in all these sections due to its length.

Q3. *The sample selected from each of the 17 countries should be clearly written in the paper.*

R3. As mentioned above, we re-wrote the Methods section to make this clearer. Additionally, we provide more details about the sample sizes for each country in the Supplementary Information section. Supplementary Table S1 shows the total sample sizes by country along with the geographic coverage of our data within each country. This table is reproduced below for your convenience:

Table S1. Date of Launch and Number of Observations by Country

Country	(1) Launch Date	(2) No. of Observations	(3) % of Localities with Observations
Chile	3/27/2020	35,556	97%
Bolivia	4/1/2020	25,970	83%
Panama	4/3/2020	15,521	100%
Uruguay	4/3/2020	21,191	64%
Peru	4/7/2020	25,452	47%
Mexico	4/13/2020	19,483	51%
Costa Rica	4/13/2020	9,151	90%
Colombia	4/15/2020	23,458	71%
El Salvador	4/16/2020	13,456	50%
Dominican Republic	4/16/2020	7,965	94%
Barbados	4/16/2020	2,072	100%
Jamaica	4/16/2020	2,547	91%
Guyana	4/16/2020	1,670	96%
Bahamas	4/16/2020	896	81%
Trinidad and Tobago	4/16/2020	4,683	100%
Ecuador	4/16/2020	18,688	68%
Suriname	4/17/2020	565	100%

Date of launch is the date on which the social media posts began. The date that the survey was rolled out in each country was largely determined by bureaucratic processes and approvals. With the except of Costa Rica, data collection in each country continued until April 30, 2020. The number of observations for each country reflected the number in the sample after data cleaning. The percent of localities is the percent of localities of each country for which we have one or more observations in the sample.

Q4. *Please provide additional information on how the data was combined and analyzed to support the results.*

R4. In the Methods section we added a new subsection "Statistical Analysis" that describes how the data was combined and analyzed for the analysis. This section is reproduced below for your convenience:

> "The study presents descriptive statistics for the relevant outcomes aggregated across countries (e.g., rates of job loss and business closure). Details on the analyses conducted for each figure are available in the Supplementary Information. The

study further examines how the loss of livelihood relates to changes in household nutrition and policy support by estimating linear regressions. These linear regressions include various controls to isolate time-varying locality shocks and prevent differences in industry sectors from driving results. Refer to the Supplementary Information section for more details on the estimations."

Details for the estimations presented in each of the figures and tables are provided in the Supplementary Information section, in the subsection titled "Statistical Analysis Details" to keep the main article concise.

Q5. *Standard English should be used to write the overall paper. As the written English is not clear, so it is difficult to understand the paper properly.* ***[note: we combined Q5 and Q6 because they referred to the same issue]***

R4. We restructured the entire article which we believe improved the flow and readability of the paper. In addition, we sent the paper to a professional copyeditor. We believe that after implementing these changes, the paper is now written in clear standard English and is easy to understand properly.
